# Hydroxyapatite as an Enzyme Carrier in Continuous-Flow Biocatalysis: An Improved Synthesis of γ‑Glutamyl Derivatives

**DOI:** 10.1021/acs.oprd.6c00083

**Published:** 2026-06-19

**Authors:** Leonardo Gelati, Fabrizio Medici, Sebastiano Campisi, Cinzia Calvio, Maurizio Benaglia, Antonella Gervasini, Marco Rabuffetti, Giovanna Speranza, Carlo F. Morelli

**Affiliations:** † Dipartimento di Chimica, 9304 Università degli Studi di Milano, via Golgi 19, 20133 Milan, Italy; ‡ Dipartimento di Architettura e Disegno Industriale, Università degli Studi della Campania Luigi Vanvitelli, via San Lorenzo − Abazia di San Lorenzo, 81031 Aversa, Italy; § Dipartimento di Biologia e Biotecnologie, Università degli Studi di Pavia, via Ferrata 9, 27100 Pavia, Italy

**Keywords:** hydroxyapatite, enzyme immobilization, γ-glutamyltransferase, continuous-flow reaction γ-l-glutamyl-*S*-allyl-l-cysteine, enzyme reusability

## Abstract

Biocatalysis has proved to be of great importance to pursue a more sustainable production of fine chemicals, and enzyme immobilization is a crucial tool to achieve this goal. In this work, hydroxyapatite, an eco-friendly material, was used as a support for the immobilization of a γ-glutamyltransferase from *Escherichia coli*, an enzyme that catalyzes the synthesis of bioactive γ-glutamyl derivatives, exploiting the noncovalent interactions between the support and the enzyme. After screening the immobilization conditions, the storage (up to 90 days) and the thermal stability (50 °C) of the obtained immobilized biocatalyst were studied, as well as its reuse in up to 10 consecutive reactions of γ-glutamylation of *S*-allyl-l-cysteine, to give γ-l-glutamyl-*S*-allyl-l-cysteine (**3**), a flavor enhancer occurring in garlic extract. Finally, the immobilized enzyme was used to prepare a packed-bed reactor and **3** was synthesized under continuous-flow conditions improving both the productivity (from 19.4 to 35.1 μmol·h^–1^) and the space-time yield (9.7 vs 381 μmol·h^–1^·mL^–1^) of the reaction.

## Graphical abstract

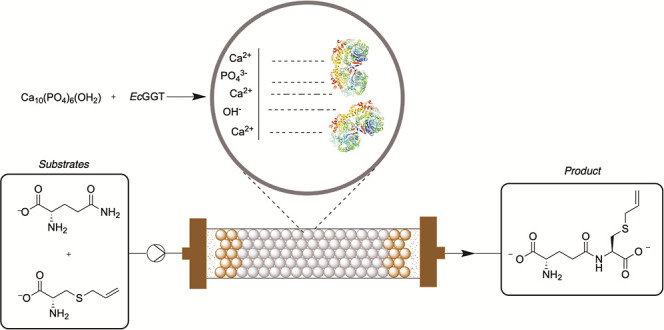

## Introduction

The past three decades have seen a transformative shift of chemical industry toward more sustainable practices based on the principles of green chemistry.^1–3^ In particular, a number of strategies have been adopted for reducing the use and generation of hazardous substances, thereby minimizing the environmental impact of chemical processes.^4^ Among them, biocatalysis, i.e., the use of enzymes in organic synthesis, has been recognized as a key enabling technology in many industrial sectors as it also answers the increasing demand for safe and highly selective processes carried out under mild reaction conditions that ensure reduced energy consumption and lower process costs.^5^ Thus, enzyme-based catalysis offers both economic and environmental advantages over traditional chemical catalysis and entirely fits into the principles and metrics of green chemistry.^6^

Frequently, in biocatalyzed processes, enzymes are not used in their native form: especially for large-scale industrial applications, it is more advantageous to use them in the form of water-insoluble powders, i.e., attached to a solid support.^6,7^ Therefore, in the recent decades, enzyme immobilization has evolved into a broadly applied tool for chemical synthesis involving biocatalysis.^8,9^ Such a methodology addresses the two main limitations associated with the use of free enzymes in organic synthesis, i.e., their reusability and their low stability in the reaction conditions.^10^

Indeed, the binding of an enzyme molecule to an insoluble support turns it into a solid heterogeneous catalyst. Thus, it can be easily recovered from the reaction mixture by simple filtration or centrifugation and then recycled, resulting in better reaction control, simplification of the process, improvement of the product quality, and reduction of the environmental footprint.^11^ Moreover, the bonds between the carrier and the biocatalyst usually make the quaternary structure of the protein more rigid, decreasing its tendency to denature under adverse conditions, thus increasing its operational stability.^12,13^

Enzyme immobilization also enables biocatalysts to be integrated into continuous-flow systems, which offer a number of benefits, such as enhanced mass and heat transfer, improved control of the reaction parameters, and in-line purification. Hence, flow biocatalysis, i.e., the use of enzymes in continuous flow, has emerged as a powerful and sustainable strategy to improve the performance of enzyme-mediated transformations.^14,15^

In immobilizing an enzyme, the choice of the support is of paramount importance, since it can greatly affect the catalytic efficiency of the biocatalyst.^16^ Some features are common to most of the materials used as enzyme carriers, namely, insolubility, chemical, mechanical, and thermal stability under operational conditions, resistance to microbial attack, affinity to the enzyme, large surface area, suitable particle size, presence of functional groups that can be exploited for the immobilization process, and, given the strong push toward circular economy, reusability.^17^

Commercially available synthetic polymers such as methacrylic resins are, in general, a first choice as enzyme supports.^7^ However, their high costs and environmental impact are significant drawbacks, especially on an industrial scale.^18,19^ Moreover, the increasing demand for more sustainable and cost-effective supports has prompted the research for materials derived from renewable resources as they represent attractive candidates to replace the petrol-based ones.^20–22^

Calcium hydroxyapatite (HAP, with formula Ca_10_(PO_4_)_6_(OH)_2_) can be enumerated among these alternatives thanks to its high insolubility in water (*K*_*PS*_ ≈2.34 × 10^–59^), its high surface area (around 100 m^2^·g^–1^), its chemical and thermal stability, and its affinity for proteins, along with the presence of functional groups suitable for surface derivatization. Moreover, its biocompatibility is granted by the fact that it is the mineral component in mammals’ bone tissue,^23^ and its “eco-friendliness” is ensured by the possibility of obtaining it from natural resources such as eggshells, fish bones, and seaweed.^24^

Although the first studies on HAP as an enzyme carrier date back to the 90s’ ^25–27^ and several examples of its use have been reported, both through adsorption and covalent binding, the potential of this eco-friendly material for enzyme immobilization has not yet been fully exploited. In particular, it has never been used as an enzyme support in continuous-flow reactors.^28^

We report here the immobilization of γ-glutamyltransferase from *Escherichia coli* (GGT, E.C. 2.3.2.2) on bare synthetic HAP through adsorption and application of the resulting heterogeneous biocatalyst in continuous-flow synthesis.

GGTs are a family of enzymes that catalyzes the transfer of a γ-glutamyl residue from a donor substrate, such as glutathione or glutamine, to an acceptor bearing a nucleophilic group.^29^ GGTs are widely distributed in all living organisms, from bacteria to mammals, and play vital roles in various cellular processes.^30^ In addition, GGTs have been used both in free and immobilized forms for the synthesis of γ-glutamyl peptides,^29,31,32^ a class of naturally occurring molecules frequently endowed with biological activity.

In this work, GGT from *Escherichia coli* (*Ec*GGT) was immobilized on bare HAP through adsorption both in batch and in flow conditions and used to perform reactions in continuous-flow mode. In particular, the synthesis of γ-l-glutamyl-*S*-allyl-l-cysteine (**3**), a flavor enhancer with kokumi properties (Scheme 1), was successfully carried out. To the best of our knowledge, this is the first example of the use of HAP as an inexpensive and renewable carrier in continuous-flow biocatalysis.

**Scheme 1:**
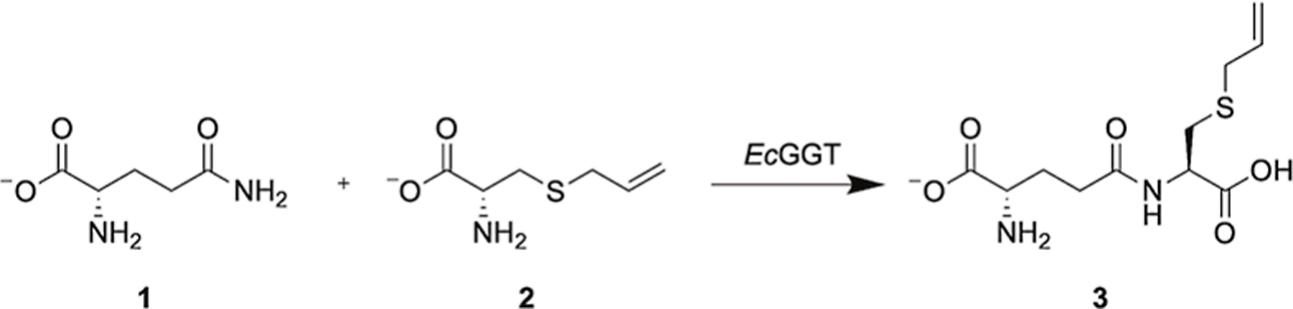
Synthesis of γ-l-Glutamyl-*S*-allyl-l-cysteine (**3**) Catalyzed by *Ec*GGT

## Materials and Methods

### General Information

Calcium nitrate tetrahydrate, ammonium phosphate dibasic, allyl bromide, l-glutamine, l-glutamic acid, l-serine, l-cysteine hydrochloride monohydrate, l-glutamic acid 5-(*p*-nitroanilide) (GPNA), glycylglycine (GlyGly), and 1-fluoro-2,4-dinitrobenzene (Sanger’s reagent) were procured from Merck KGaA (Darmstadt, Germany). All reagents were used as received without further purification. HPLC-grade solvents were obtained from Aldrich.

GGT from *E. coli* was obtained as previously described.^33^

Experiments with hydroxyapatite of particle sizes between 180 and 250 μm were performed in a round-bottom flask stirred using a rotary evaporator apparatus.

Continuous-flow experiments were carried out using the ASIA pump system (Syrris Ltd., Royston, UK).

Analytical TLC was performed on silica gel F254 precoated aluminum sheets (0.2 mm layer) (Merck, Darmstadt, Germany). The eluent was a mixture of *n*-BuOH/water/AcOH 4:1:1. Detection: 5% w/v ninhydrin solution in ethanol, followed by heating at 150 °C ca.

HPLC analyses were carried out using a 250 × 4.6 mm Gemini RP C18 column (Phenomenex, Torrance, CA, USA) on a Jasco instrument equipped with an UV/vis detector. Eluent A was 0.1% trifluoroacetic acid; eluent B was an 80:20 mixture of acetonitrile and eluent A. The following gradient was used: 0–10 min, isocratic elution with eluent A:eluent B 80:20; 10–15 min, linear gradient to eluent A:eluent B 70:30; 15–25 min, linear gradient to eluent A:eluent B 50:50; 25–35 min, linear gradient to eluent A:eluent B 40:60; 35–40 min, isocratic elution with eluent A:eluent B 40:60; 40–60 min, column re-equilibration through linear gradient to eluent A:eluent B 80:20. The flow rate was 1 mL·min^–1^ and detection was at 356 nm.

Ion exchange column chromatography was performed with Dowex 1 × 8 resin 200–400 20 mesh (Aldrich, Darmstadt, Germany) in the acetate form.

1H NMR spectra were acquired at 400.13 MHz on a Bruker Advance 400 spectrometer (Bruker, Karlsruhe, Germany) interfaced with a workstation running Windows operating system and equipped with a TOPSPIN software package. Chemical shifts are given in ppm (δ) and are referenced to solvent signal (δH D_2_O 4.79 ppm). Spectra analyses were carried out with TOPSPIN software.

ATR-FTIR spectra were recorded with a Jasco FT/IR-4600 spectrophotometer (Jasco International, Tokyo, Japan).

SEM images were obtained by using an FESEM Sigma microscope (Zeiss).

XRPD analyses were carried out with a PANanalytical X’Pert PRO diffractometer (Cu Kα = 1.54060 Å) equipped with an X-ray source operating at 40 kV × 40 mA.

Measurements of surface area and pore size were performed by nitrogen adsorption–desorption using a Carlo Erba Sorptomatic 1990 instrument.

UV measurements were carried out with a Jasco V-360 spectrophotometer (Jasco International, Tokyo, Japan).

### Hydroxyapatite Synthesis

The synthesis of hydroxyapatite (HAP) was performed by following a literature-reported method.^34^

Briefly, (NH_4_)_2_HPO_4_ solution (0.1 M in degassed Milli-Q water, 250 mL) was placed in a five-necked 1 L round-bottom flask under a nitrogen atmosphere and heated to 80 °C and NH_4_OH (30%, 50 mL) was added. Then, a Ca­(NO_3_)_2_·4H_2_O solution (0.167 M in degassed Milli-Q water, 250 mL) was added under stirring through a peristaltic pump (flow rate 1.67 mL·min^–1^). A white solid started to precipitate.

Every 30 min, NH_4_OH (30%, 10 mL) was added to maintain the pH basic. The reaction was stirred at 80 °C for 2.5 h and then left aging under stirring for 5 min.

Hot filtration under reduced pressure was followed by washes with hot Milli-Q water until the filtrate reached a neutral pH (ca. 4 × 500 mL). The obtained white solid was dried at 70 °C under reduced pressure (around 500 mbar) for 16 h and then at 120 °C for 8 h. The dried solid was ground to obtain a homogeneous particle size leading to a white powder.

To obtain larger particle sizes, the powder was first pressed using a hydraulic press; the resulting wafer was then ground and sieved using woven wire mesh stainless steel sieves by Endecotts, to obtain homogeneous particles in the size range of 180–250 μm.

### GGT Activity Assay

20 μL of enzyme solution was added to a 1.980 mL solution of 1 mM GPNA and 100 mM GlyGly in 100 mM TRIS buffer at pH 8.5 at room temperature. The release of *p*-nitroaniline was continuously monitored at 410 nm, registering data every 10 s for 180 s. One enzyme unit was defined as the amount of enzyme that liberates 1 μmol·mL^–1^·min^–1^ of *p*-nitroaniline. The concentration of *p*-nitroaniline was determined through a calibration curve (Figure S1). All the measurements were performed in triplicate.

### GGT Immobilization Condition Screening

The determination of favorable immobilization conditions was performed as follows: hydroxyapatite (50 mg) of different particle sizes, namely, <200 nm or 180–250 μm, was suspended in an appropriate medium (100 mM carbonate/bicarbonate buffer (CBB), 100 mM Tris, or deionized water) in the amount needed to have a final volume of 1.0 mL, at a certain pH (between 7 and 9.2) and thermostated at the desired temperature (0 or 30 °C). GGT from *Escherichia coli* (0.30 U, final supernatant activity 0.30 U·mL^–1^) was added and the suspension was stirred for 4 h.

The immobilization process was monitored by taking an aliquot (40 μL) of supernatant every hour and performing the enzymatic activity assay on it.

The mixture was then separated by centrifugation (8000 rpm, 2 min, Universal 32 Hettich Zentrifugen) in the case of nanometric particles or by filtration under reduced pressure in the case of micrometer ones. The solid was washed with the immobilization medium (2 × 1 mL).

The enzyme concentration in both the filtrate and the washing was measured through Bradford assay, and the residual activity was determined by activity assay. The final immobilization yield (IY) was determined as reported in the Supporting Information, paragraph 1.5.4.

### Determination of the Activity of the Immobilized GGT

The GGT immobilized on HAP (180–250 μm particle size) was placed in a round-bottom flask and suspended in Tris buffer (100 mM, pH 8.5, 1.0 mL). GlyGly (200 mM in TRIS pH 8.5, 2.0 mL) and GPNA (4 mM in Tris pH 8.5, 1.0 mL) solutions were added and the reaction was stirred for 25 min at room temperature. Ten times periodically (namely, after 1, 2, 3, 4, 7, 9, 12, 15, 20, and 25 min), the supernatant (40 μL) was taken and diluted 1:20 in Tris pH 8.5 and the absorbance at 410 nm was measured. From the obtained data, it was possible to determine the activity of the immobilized GGT.

### Immobilized GGT Stability

The immobilized GGT stability was tested against different desorbing agents and different temperatures. Moreover, its storage stability at 4 °C was evaluated over a 90 day period. For more details, see paragraph 1.6 in the Supporting Information.

#### Enzymatic Synthesis of γ-l-Glutamyl-*S*-allyl-l-cysteine (**3**) and Enzyme Recyclability

l-Glutamine (Gln, 73.1 mg, 0.50 mmol) and *S*-allyl-l-cysteine (SAC, 80.6 mg, 0.50 mmol) were dissolved in deionized water and the pH was adjusted to 10 using an aqueous NaOH solution (1.0 M, final volume of the solution 5.0 mL). 2 mL of the resulting solution was added to the immobilized GGT (50 mg, 0.32 U). The reaction mixture was thermostated at 30 °C and stirred for 7 h. Every hour, the supernatant (20 μL) was taken and derivatized for HPLC analysis (see paragraph 1.7.3 of the Supporting Information).

After 7 h, the supernatant was removed with a Pasteur pipet and the immobilized enzyme was washed with Tris buffer (0.1 M, pH 8.5, 2 × 1 mL). The enzyme was stored at 4 °C until the next reaction cycle. The enzyme was recycled 9 times.

### Continuous-Flow Adsorption

A solution of GGT in Tris buffer (100 mM, pH 8.5) was recirculated through the packed-bed reactor (flow rate 250 μL·min^–1^) at room temperature. Different enzymatic activities (between 0.036 and 0.25 U·mL^–1^) and cycle numbers (6 or 8) were tested. The adsorption process was monitored by performing the enzymatic activity assay on the recirculating solution.

After the immobilization was completed, the packed-bed reactor was washed with Tris buffer (100 mM, pH 8.5).

### In-Flow Synthesis of γ-l-Glutamyl-*S*-allyl-l-cysteine (**3**)

A solution containing l-glutamine (**1**, 100 mM) and *S*-allyl-l-cysteine (**2**) (either 100 mM or 300 mM) in NaOH_(aq)_ (pH 10) was flowed through the packed-bed reactor (2.5 U·g^–1^) with a residence time of 10 min at 30 °C. For each reaction, six reaction volumes were collected and the last three were derivatized and analyzed by HPLC.

## Results and Discussion

### Hydroxyapatite Synthesis and Characterization

Hydroxyapatite used for immobilization of *E. coli* GGT was synthesized by coprecipitation under wet conditions as previously reported.^34^

The obtained material was characterized by attenuated total reflection Fourier-transform infrared spectroscopy (ATR-FTIR), X-ray powder diffraction (XRPD), scanning electron microscopy (SEM), and nitrogen adsorption–desorption curves (BET method).

The XRPD pattern of the bare support (Figure S2a) confirms the unique presence of the hydroxyapatite phase (JCPDS: 00-09-0432) with a hexagonal crystal structure. The characteristic diffraction peaks are observed at 2θ values of 25.9°, 28.1°, 31.8°, 32.8°, 39.9°, 46.7°, and 49.5°, which can be indexed to the (002), (012), (211), (300), (310), (222), and (320) planes, respectively. All peaks are sharp, indicating a high degree of crystallinity in the hydroxyapatite sample. Its FTIR spectrum (Figure 1, orange line) clearly shows the expected vibrational bands of phosphate, carbonate, water, and hydroxyl groups.^35–38^ The phosphate signals are well-defined, with ν4 bending at 558 and 600 cm^–1^, ν_1_ symmetric stretching at 963 cm^–1^, and intense ν_3_ antisymmetric stretching at 1020 and 1089 cm^–1^. Carbonate is also present, as commonly observed in both synthetic and biological apatites, and appears in two forms: B-type, where carbonate substitutes phosphate groups (1420 cm^–1^), and A-type, where carbonate replaces hydroxyl groups in the lattice channels (1454 cm^–1^). A combined contribution of both types is also visible at 1493 cm^–1^, while the ν_2_ out-of-plane bending at 881 cm^–1^ further confirms carbonate incorporation. Bands related to adsorbed water (1648 cm^–1^) and columnar hydroxyl groups (3569 cm^–1^) are also evident. In addition, the SEM images (Figure S3a) showed that HAP particles have irregular shapes and a rough surface with a size distribution in the range 180–250 μm. Finally, the HAP sample showed a large surface area (87 ± 5 m^2^·g^–1^ determined by BET analysis) with a pore volume of 0.22 ± 0.02 m^3^·g^–1^.

**Figure 1:**
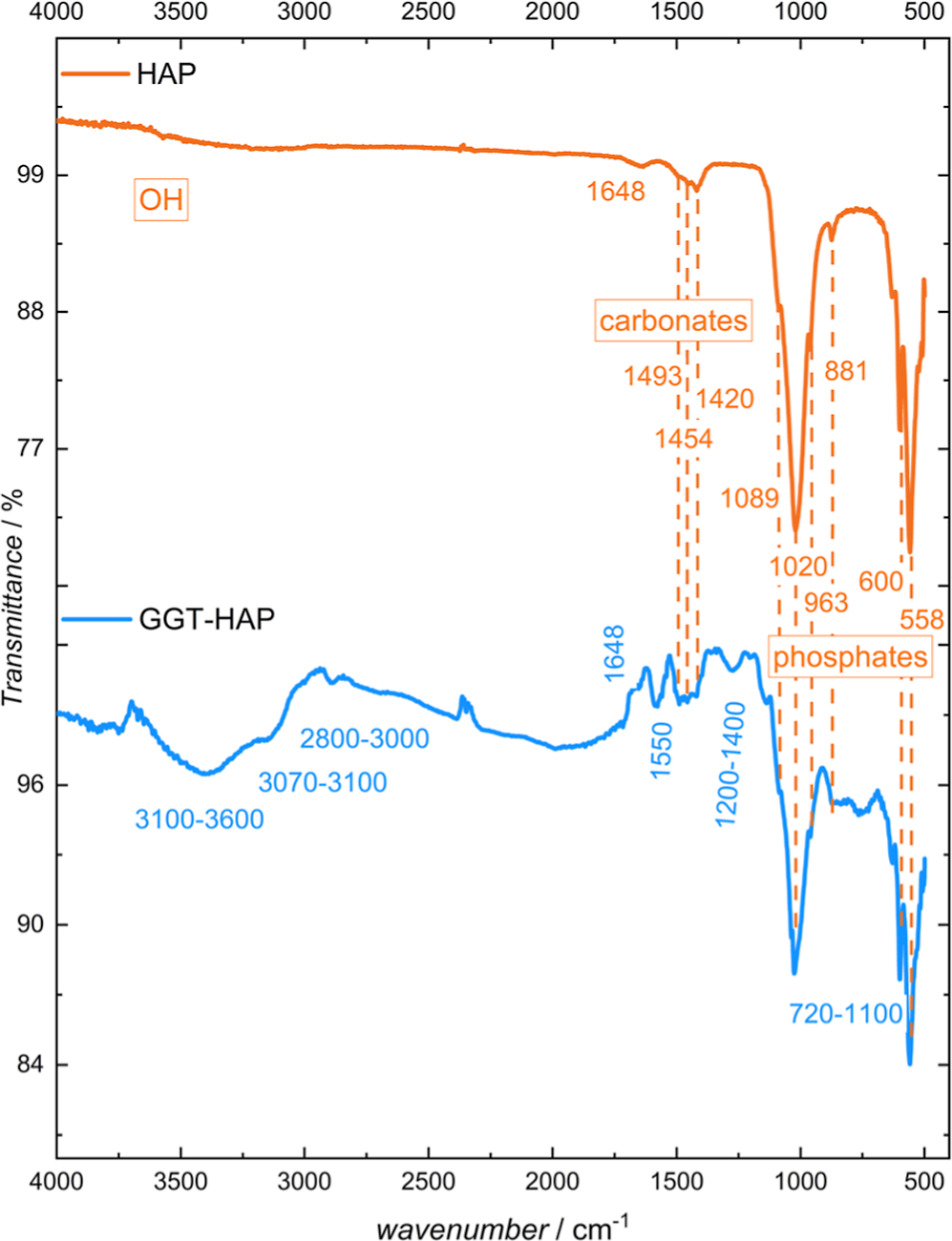
Comparison of ATR-FTIR spectra of pristine hydroxyapatite (HAP, orange line) and the material after GGT immobilization (GGT-HAP, cyan line).

### Evaluation of the Immobilization Conditions

Taking into account the preferred conditions for GGT-catalyzed reactions and the stability range of HAP, as a first approach to determine the ideal conditions for GGT immobilization, the effect of different parameters, i.e., reaction medium, pH, temperature, and support particle size, was evaluated.

The immobilization time was set to 4 h (see Supporting Information for details), an enzyme concentration of 0.3 U·mL^–1^, corresponding to a maximum enzyme loading of 6.0 U·g^–1^, was used, and pH was varied between 7 and 9.2 according to the optimal activity of the enzyme.^39^ Using water as a reference medium, a high immobilization yield (IY) could be obtained by performing the adsorption both at 0 and 30 °C (Table 1, entries 1–4). By contrast, carbonate/bicarbonate buffer (CBB), frequently used as a reaction medium in GGT-mediated biotransformations,^40^ did not allow the adsorption to happen, independently of the temperature and the particle size (entries 5–8).

**Table 1:** Condition Screening for the Adsorption of GGT on HAP

entry	particle size	solvent	pH	temperature	IY^a^
1	<200 nm	water	7	0 °C	97%
2	<200 nm	water	7	30 °C	94%
3	180–250 μm	water	7	0 °C	74%
4	180–250 μm	water	7	30 °C	87%
5	<200 nm	CBB^b^	9.2	0 °C	0%
6	<200 nm	CBB^b^	9.2	30 °C	0%
7	180–250 μm	CBB^b^	9.2	0 °C	0%
8	180–250 μm	CBB^b^	9.2	30 °C	0%
9	<200 nm	Tris^c^	9	0 °C	82%
10	<200 nm	Tris^c^	9	30 °C	80%
11	180–250 μm	Tris^c^	9	0 °C	34%
12	180–250 μm	Tris^c^	9	30 °C	73%
13	180–250 μm	Tris^c^	8.5	0 °C	91%
14	180–250 μm	Tris^c^	8.5	30 °C	96%

a Determined by measuring the residual activity in the supernatant.

b 100 mM Carbonate/Bicarbonate Buffer.

c 100 mM Tris buffer.

Experiments performed in Tris buffer, the medium of choice to perform the GGT activity assay, afforded from modest to very high immobilization yields (up to 96%), depending on pH and temperature (entries 9–14). The latter has a high impact on the adsorption process only at pH 9 when HAP particles have a size between 180 and 250 μm (entries 11 and 12), the immobilization yields (IY) being less affected by smaller size particles (entries 9 and 10) and a slight decrease in pH (pH 8.5, entries 13 and 14).

However, HAP with particle sizes between 180 and 250 μm was selected for all the further experiments, as nanometric HAP (<200 nm) was found to be difficult to handle both in batch and in flow conditions (see below). For example, nanometric HAP remained dispersed in the medium for several minutes after stirring was stopped, making it difficult to measure enzyme activity correctly.

The effect of GGT loading on the outcome of the immobilization process was also investigated. Data from Table 2 indicate that there was a small decrease in the immobilization yield with the increase of the enzymatic load offered (from 2.5 U_GGT_ g^–1^_support_ up to 4-fold higher), while the amount of immobilized units, expressed as efficiency and activity recovery, presented a significant drop. This can be due to the fact that the support area is sufficient to accommodate most of the enzyme molecules offered, but the excessive enzyme loading might cause protein–protein interactions that limit the flexibility of the biocatalyst, leading to a loss in activity.^41,42^

**Table 2:** Effect of GGT Loading on the Immobilization Outcome Process

entry	enzyme loading (U·g^–1^)	IY (%)	efficiency (%)	activity recovery (%)
1	2.5	95	84	81
2	5.0	87	97	85
3	10	88	49	43

### GGT-HAP Characterization

To confirm the success of the immobilization process, the GGT-HAP derivative was characterized with the same techniques used for the pristine support.

No significant differences were found between samples of pure HAP and GGT-HAP by XRPD analysis, suggesting that the enzyme is adsorbed on the support surface without substantially affecting its crystalline structure (Figure S2b). As for the FTIR spectrum, after immobilization of the enzyme (Figure 1, cyan line), the phosphate region remains unchanged, confirming that the crystal structure of HAP is preserved. In the carbonate and hydroxyl regions, however, some overlap with enzyme-related vibrations, such as amide backbone and side-chain modes, can be observed.^43,44^ Among signals characteristic of peptide bonds, it is possible to identify the amide III band (C–N stretching and N–H bending, between 1200 and 1400 cm^–1^), the amide II (N–H bending and C–N stretching at 1550 cm^–1^), the amide I (predominantly C=O stretching of the peptide backbone at 1650 cm^–1^), and N–H stretching signals (amide B at 3070–3100 cm^–1^ and amide A between 3100 and 3600 cm^–1^). These additional protein-related signals indicate that GGT enzyme was successfully immobilized without significant modifications of the HAP structure (see Table S1 for assignments of the most prominent bands).

Finally, SEM images (Figure S3b) after GGT absorption showed that the particles have a smoother surface than pure HAP. This feature was already observed by Ding et al. for dextranase adsorbed on HAP exploiting either Tris buffer or acetate buffer as the reaction medium.^45^

### Desorption Tests

To gain insight into the type of enzyme–support interaction, a number of experiments in the presence of potential desorbing agents were performed, varying both the type of desorbing agent and its concentration. As can be seen in Table 3, 100 mM CBB not only showed adverse effects in the adsorption process but also caused a high degree of desorption (60%) of the immobilized GGT. Also in the case of NaCl, a relevant and concentration-dependent desorption of the enzyme was observed, suggesting that in order to disrupt the ionic interactions between GGT and the support, high concentrations of this salt are required.

**Table 3:** Desorption of GGT from HAP

entry	solvent	pH	desorbed protein (%)
1	CBB (100 mM)	9.2	60
2	NaCl (100 mM)	7.0	30
3	NaCl (200 mM)	7.0	34
4	NaCl (500 mM)	7.0	41
5	NaCl (1 M)	7.0	45
6	Tris (100 mM)	8.5	4
7	sodium citrate (200 mM)	8.3	67
8	sodium citrate (500 mM)	8.3	92

Conversely, immobilized GGT was desorbed only in a small amount (4%) in the presence of 100 mM TRIS, pH 8.5. The strong physical adsorption of GGT onto HAP is of relevance, indicating that the enzyme cannot be leached from the support under the selected adsorption conditions.

Finally, sodium citrate solution was found to have the highest desorbing capacity, resulting in almost complete removal (92%) of the enzyme from the support at 500 mM concentration. The strong desorbing effect of this tricarboxylic acid indicates that the key factor in the adsorption process is the interaction of the calcium ions on HAP with the COO^–^ groups of aspartic and glutamic residues on the enzyme surface.^42,45,46^ A similar effect could be responsible for the desorption ability of CBB: indeed, the carbonate ions of such a buffer can compete with the acid residues of the GGT in the chelation of the superficial Ca^2+^ ions of the support surface.

### Thermal and Storage Stability

One of the advantages of enzyme immobilization is the increased stability of the biocatalyst under adverse reaction conditions, such as temperatures higher than those tolerated by the free enzyme. Thus, GGT from *E. coli*, both in the free and adsorbed form, was incubated for one hour at different temperatures, namely, 30, 40, and 50 °C, and its activity was then tested. Considering the GGT activity measured at 30 °C as 100%, profiles reported in Figure S4 show that after heat treatment at 40 and 50 °C, the immobilized GGT retained around 90% and 20%, respectively, of the original activity, while the residual activity of the free enzyme remained constant at 40 °C and dropped below 10% after incubation at 50 °C. Although the preserved activity of GGT-HAP was modest, this result confirmed that the enzyme becomes more thermostable when immobilized on HAP.

The storage stability of the immobilized enzyme at 4 °C was also monitored and compared to that of the free GGT (Figure 2, cyan and orange bars, respectively). In both cases, after a slow decrease within the first 3 weeks, the enzymatic activity remained fairly stable, at around 50% of the initial one, up to 90 days. The fact that the stability of GGT-HAP presents a similar trend to that of free GGT suggests that the weak interactions for the physical adsorption of the enzyme do not provide any improvement of its stability over time.

**Figure 2:**
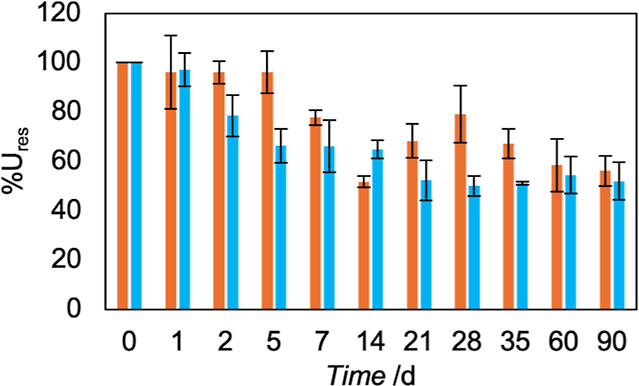
Stability of the free (orange bars) and immobilized (cyan bars) GGT after storage at 4 °C.

Covalent immobilization could be a viable possibility for increasing the stability of the immobilized enzyme. Some strategies for HAP functionalization and subsequent enzyme covalent immobilization have been recently reported,^47^ and starting from those data, a systematic study for the covalent immobilization of GGT is currently ongoing in our laboratories.

### Enzyme Reusability

Two further advantages of using immobilized enzymes are both the easy recovery of the biocatalyst from the reaction mixture, thus permitting its reuse in consecutive reactions, and the possibility of implementing biocatalysis in continuous-flow synthesis.

As a first approach, the reusability of GGT adsorbed on hydroxyapatite was evaluated by performing five consecutive activity assays (Figure 3a). Considering the initial enzyme activity as 100%, a decreased enzyme efficiency was observed in the second cycle (ca. 65%), after which the immobilized GGT maintained about 50%–60% of its original activity for the next four cycles. In addition, the recyclability of the biocatalyst was tested in consecutive batches of γ-glutamylation of *S*-allyl-l-cysteine (SAC, **2**) carried out under the same conditions as for the free enzyme. Reactions were performed at 30 °C on an analytical scale (0.2 mmol) in pure water, and the pH was adjusted to 10 with 1 M NaOH.

**Figure 3:**
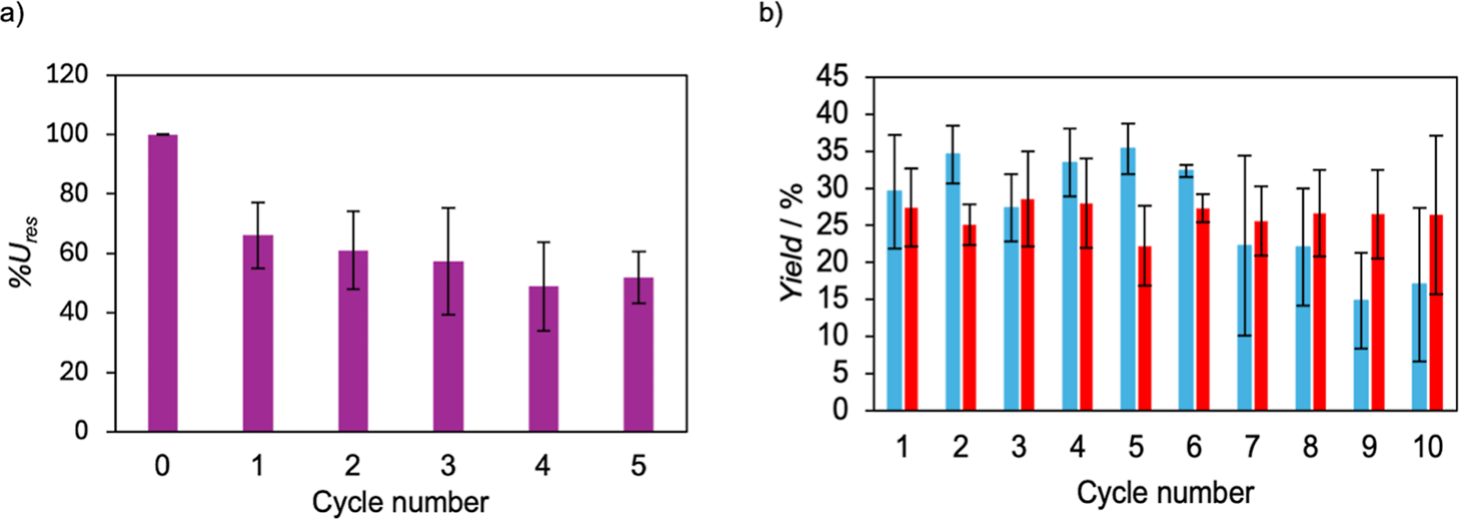
Recyclability of the immobilized GGT in (a) multiple consecutive activity assays and (b) multiple γ-glutamylations of SAC (**2**) in batch (cyan bars) and in continuous flow (red bars).

It is worth noting that the GGT-mediated transfer of a γ-glutamyl moiety from a donor substrate, such as glutamine (**1**), to an acceptor, such as SAC (**2**), suffers from rather low yields due to the presence of competing reactions, i.e., autotranspeptidation and hydrolysis reactions producing γ-glutamylglutamine and glutamic acid, respectively (see Scheme S1).^40^ This outcome is due to the mechanism of GGT-catalyzed reactions: in the first step, the donor (e.g., Gln) transfers its glutamyl moiety to a threonine residue in the active site of the enzyme, generating a γ-glutamyl–enzyme intermediate. The latter is susceptible to nucleophilic attack and, depending on the nature of the nucleophile (either the acceptor or another molecule of the donor or water), can lead to different products. Indeed, when the free enzyme was used, a 34% HPLC yield was achieved, in agreement with previously reported results.^48^ Also, GGT-HAP led to similar results (ca. 30% HPLC yield), but the immobilized biocatalyst could be reused up to 10 consecutive reactions without any major loss of reactivity up to the sixth reaction cycle (Figure 3b, cyan bars).

Thus, the possibility of reusing the biocatalyst in multiple consecutive reactions allowed the synthesis of a higher amount of γ-l-glutamyl-*S*-allyl-l-cysteine (**3)** per unit of enzyme with respect to that obtained using the free enzyme in homogeneous conditions, even if the latter requires lower reaction times (3 h vs 7 h, see the Supporting Information). The turnover number (TON) of the immobilized GGT in the first reaction cycle, calculated as reported in ref 49, was 2.8 × 10^4^, while the cumulative TON over the ten reactions resulted to be 2.6 × 10^5^.

### Reaction Condition Optimization and Scale-up

With the aim of optimizing the γ-l-glutamyl-*S*-allyl-l-cysteine (**3**) synthesis, the SAC (**2**) concentration was increased in order to drive the equilibrium of the reaction toward the transpeptidation product. In particular, following a previous report,^48^ the SAC (**2**)/glutamine (**1**) ratio was modified. At first, the acceptor/donor molar ratio was increased to 2:1, leading to an HPLC yield of ca. 52% after 7 h. The yield was improved up to ca. 68% when a molar ratio of 3:1 was used. In such optimized conditions, the reaction was performed on a semipreparative scale (1 mmol) and monitored by HPLC analyses (66% yield after 7 h). The product was isolated by ion exchange chromatography in 51.2% yield.

### Continuous-Flow Adsorption

Immobilized enzymes can be combined with continuous-flow technology to increase reaction productivity and make the enzyme reuse easier.^50,51^ In order to achieve this, GGT from *Escherichia coli*, dissolved in Tris buffer (100 mM, pH 8.5), was adsorbed on hydroxyapatite packed in an Omnifit column, to generate a so-called packed-bed reactor. In order to avoid column filter clogging caused by leaching of the support, the HAP (180–250 μm) layer was placed between two layers of sand. After evaluating the void volume of the reactor, by flowing through it a *p*-nitroaniline solution (1.5 mM in Tris, pH 8.5), the GGT solution was recirculated multiple times through the reactor at a constant flow rate of 250 μL·min^–1^.

Different enzymatic activities and loading were tested. In all of the cases, as reported in Table 4, enzyme adsorption was almost quantitative after just a few cycles. Although the continuous-flow biocatalyzed synthesis of **3** mediated by a different GGT from *Bacillus subtilis* (*Bs*GGT) supported on glyoxyl agarose has already been performed,^52^ to the best of our knowledge, the direct enzyme immobilization in flow on hydroxyapatite has never been reported so far.

**Table 4:** Direct In-Flow Immobilization

entry	GGT activity (U·mL^–1^)	GGT loading (U·g^–1^)	number of cycles	IY (%)^a^
1	0.0961 ± 0.0006	2.9	6	98
2	0.0362 ± 0.0007	0.70	6	>99
3	0.1249 ± 0.0004	2.5	8	99
4	0.2498 ± 0.0005	2.5	8	98

a Determined by measuring the residual enzymatic activity in the recirculating solution.

### Residence Time Screening

The conditions reported in entry 4 of Table 4 were selected, as they conjugated fast immobilization (ca. 1 h) with good enzyme loading. Thus, a packed-bed reactor was assembled and used at flow rates in the range 4–100 μL·min^–1^ to optimize the residence time for the glutamylation reaction of SAC (**2**), monitored by HPLC analysis.

As reported in Table 5, a 10 min residence time resulted in the most favorable setting to study the packed-bed reactor reusability. In fact, by doubling the residence time, i.e., 20 min (entry 4), only a low increase of the reaction yield was obtained. On the other hand, the experiments performed with a residence time of 5 min (entry 2) afforded a similar yield, but an increase in backpressure was noticed during the run.

**Table 5:** Optimization of the Residence Time

entry	residence time (min)	flow rate (μL·min^–1^)	HPLC yield (%)
1	1	94	4
2	5	19	19
3	10	9.4	22
4	20	4.7	27

### In-Flow Recyclability

To test the robustness of the immobilized enzyme, the packed-bed reactor was used in ten consecutive glutamylation reactions of SAC (**2**) using a 1:1 reagent ratio (Scheme 2).

**Scheme 2:**
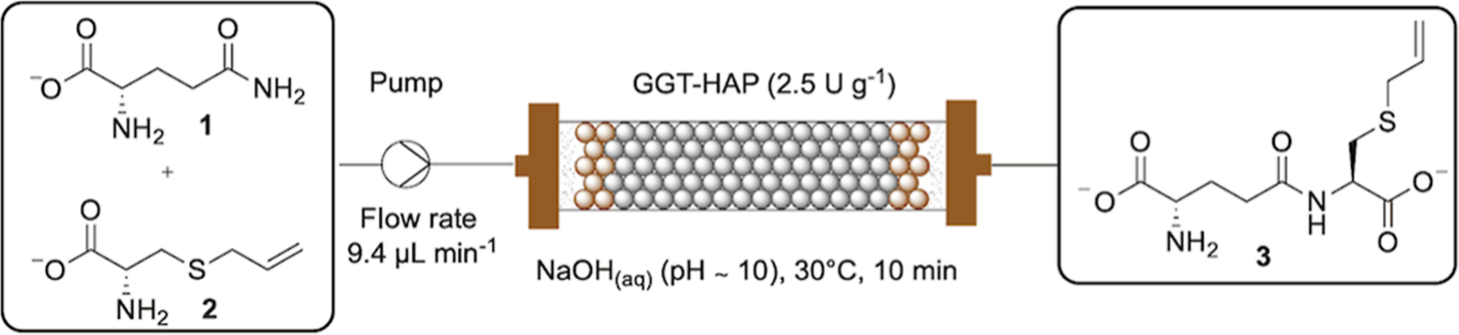
Continuous-Flow Biocatalyzed Glutamylation of *S*-Allyl-l-cysteine (**2**)

As shown in Figure 3b, a yield of around 30% was observed, which is comparable to that of the in-batch GGT-HAP-catalyzed reaction. Such a yield remained almost constant for ten consecutive reaction cycles. These findings confirm the robustness of the in-flow GGT-HAP system and indicate the absence of enzyme leaching. It is worth noting that, in contrast to the in-flow conditions, when the GGT-HAP system was used in batch, a decrease of the yield was observed after the sixth reaction (see Figure 3b).

The same flow setup was also tested under the optimal conditions for the synthesis of **3**, i.e., an acceptor/donor ratio of 3:1, obtaining a slightly lower conversion than that obtained in batch (61% vs 68% HPLC yield). This slight decrease in reaction yield could be due to an insufficient contact time between the substrates and the GGT as well as the presence of preferential stream pathways delivering the reagents to enzyme molecules. However, this lower value is broadly compensated for by the productivity advantages of the flow setup (see below).

As already stated, the continuous-flow synthesis of **3** has been previously reported by our group.^52^ In that case, after a screening of immobilization carriers and reaction parameters, **3** was obtained in 42% isolated yield using a GGT from *Bacillus subtilis* supported on glyoxyl agarose. The different results obtained can be ascribable to differences in both the immobilization support/strategy and the nature of the employed biocatalyst (*Bs*GGT *vs Ec*GGT).

### Comparison between Batch and Flow Setups

With these data in hand, it was possible to compare the two reaction setups, i.e., batch and flow, evaluating which was the most convenient in terms of both productivity and space-time yield (STY), metrics indicative of the performance of the biocatalyzed systems. Such parameters were calculated considering the first reaction cycle and using formulas of eqs 1 and 2 (see also the Supporting Information, paragraph S1.8.5 for details).

1 $$productivity=\left(n_{prod}\times \%yield\right)/\left(t_{collection}\times 100\%\right)$$

2 $$STY=\left(n_{R}\times \%yield\right)/\left(V_{R}\times t_{residence}\times 100\%\right)$$

The results obtained comparing the setups used in the nonoptimized conditions show that the flow setup is indeed more advantageous than the batch one, with a productivity of 15.4 vs 10.3 μmol·h^–1^, i.e., 1.49 times higher. An even larger increase was obtained in space-time yield (171 vs 5.2 μmol·h^–1^·mL^–1^) with the in-flow system producing 33 times more product. The beneficial effects of the flow setup were retained also under the optimized reagent ratio: in fact, the flow setup was able to produce 35.1 μmol·h^–1^, corresponding to a productivity 1.81 times higher than that obtained in batch (19.4 μmol·h^–1^). In a similar way, the space-time yield increased from 9.7 μmol·h^–1^·mL^–1^ for the batch to 381 μmol·h^–1^·mL^–1^ for the flow, i.e., an increase in the same range of the previous one (39-fold increase).

## Conclusions

Enzyme immobilization has emerged as a powerful strategy for the implementation of biocatalysis in organic synthesis, both in batch and in continuous-flow conditions. In this work, we reported the use of hydroxyapatite (HAP), a green and biocompatible material that can be obtained in agreement with circular economy principles, as a support for the immobilization of a γ-glutamyl transferase from *Escherichia coli* *.*

The chosen enzyme was directly adsorbed on the carrier surface exploiting electrostatic interactions and metal coordination. Although the obtained insoluble biocatalyst showed only a moderate increase in thermal stability with respect to the free enzyme, it was possible to reuse it up to ten times for the production of γ-l-glutamyl-*S*-allyl-l-cysteine (**3**), widely recognized for its kokumi properties. Moreover, a packed-bed reactor containing the immobilized GGT was prepared and reused in ten consecutive γ-glutamylation reactions of SAC (**2**) to give **3** with no substantial decrease in reaction yields. In addition, the flow system increased the performance with respect to the batch one in terms of both productivity and space-time yield (up to 1.81 and 39 times higher, respectively), making this approach advantageous for potential industrial applications.

However, some challenges and limitations have to be considered. Among these, the need for large HAP particles for making the handling of the immobilized GGT easier and limiting the overpressure issues in a continuous-flow setup leads to additional steps with a potential increase in the process cost. Moreover, increasing the reactor dimensions would present some challenges in the control of the internal temperature of the packed-bed reactor. To solve this last issue, a viable strategy may be the so-called numbering-up, which consists of performing the same reaction in parallel in multiple reactors. However, the possibility of directly immobilizing the enzyme in flow as reported here can result in a more controllable and reproducible process.

## Supplementary Material



## Notes

Additional experimental details, XRPD analyses, SEM images, FTIR bands assignment list, thermal stability studies, reaction mechanism, NMR spectra, and HPLC chromatogram (PDF)

## ∥

Faculty of Science and Engineering, University of Groningen, Nijenborgh 3, 9747 AG Groningen, The Netherlands

## Abbreviations

ATR-FTIR: attenuated total reflection Fourier-transform infrared spectroscopy
CBB: carbonate/bicarbonate buffer
GGT: γ-glutamyltransferase
GGT-HAP: γ-glutamyltransferase adsorbed on hydroxyapatite
HAP: hydroxyapatite
HPLC: high-performance liquid chromatography
IY: immobilization yield
JCPDS: Joint Committee on Powder Diffraction Standards
SAC: S-allyl-l-cysteine
SEM: scanning electron microscopy
STY: space-time yield
Tris: tris­(hydroxymethyl)­aminomethane buffer
XRPD: X-ray powder diffraction.
