# Supplementary material for: Hydroxyapatite as an Enzyme Carrier in Continuous-Flow Biocatalysis: An Improved Synthesis of γ‑Glutamyl Derivatives
Source: Org Process Res Dev. 2026 Jun 19;30(7):1894–902. doi: 10.1021/acs.oprd.6c00083 (PMC13436615; doi:10.1021/acs.oprd.6c00083)
Supplement: Supplementary file 1 [file op6c00083_si_001.pdf]

Supporting information

Hydroxyapatite as an enzyme carrier in continuous flow biocatalysis: an improved synthesis of γ-glutamyl derivatives

Leonardo Gelati[a,b], Fabrizio Medici[a], Sebastiano Campisi[a], Cinzia Calvio[c], Maurizio Benaglia[a], Antonella Gervasini[a], Marco Rabuffetti[a]†, Giovanna Speranza[a*], Carlo F. Morelli[a*]

[a]Department of Chemistry, Università degli Studi di Milano, via Golgi 19, 20133 Milan, Italy
[b]Department of Architecture and Industrial Design, Università degli Studi della Campania Luigi Vanvitelli, via San Lorenzo – Abazia di San Lorenzo, 81031 Aversa, Italy
[c]Department of Biology and Biotechnology, Università degli Studi di Pavia, via Ferrata 9, 27100 Pavia, Italy
†Current address: Faculty of Science and Engineering, University of Groningen, Nijenborgh 3, 9747 AG Groningen, The Netherlands

Table of contents

1. Materials and Methods 4
1.1 General information 4
1.2 Hydroxyapatite synthesis and characterization 5
1.3 GGT expression and purification 6
1.4 GGT activity assay 6
1.5 GGT immobilization 7
1.5.1 Conditions screening 7
1.5.2 Loading screening 8
1.5.3 Determination of the activity of the immobilized GGT 8
1.5.4 Calculation of immobilization parameters 8
1.6 Immobilized GGT stability tests 9
1.6.1 Desorption tests 9
1.6.2 Thermal stability 9
1.6.3 Storage stability 9
1.6.4 Enzyme recyclability (activity assay) 10
1.7 Synthesis of γ-L-glutamyl-*S*-allyl-L-cysteine (**3**) 10
1.7.1 Synthesis of *S*-allyl-L-cysteine (**1**) 10
1.7.2 Enzymatic synthesis of γ-L-glutamyl-*S*-allyl-L-cysteine (**3**) and enzyme recyclability 11
1.7.3 Pre-column derivatization procedure with Sanger's reagent 11
1.7.4 Turnover number (TON) calculation 11
1.7.5 Optimization of γ-L-glutamyl-*S*-allyl-L-cysteine (**3**) synthesis 12
1.7.6 Semipreparative synthesis of L-γ-glutamyl-*S*-allyl-L-cysteine (**3**) 12
1.8 Continuous flow immobilization and synthesis 12
1.8.1 Determination of packed-bed reactor void volume 12
1.8.2 Continuous flow adsorption 14
1.8.3 Residence time screening 14

1.8.4 In flow synthesis of γ-L-glutamyl-*S*-allyl-L-cysteine (**3**) 14

1.8.5 Packed-bed recycle 15

1.8.6 Calculation of productivity and space-time yield (STY) 15

2. Analyses of pristine HAP and GGT-HAP 16

3. Thermal stability of free *vs* immobilized GGT 18

4. Reaction mechanism of GGT-catalyzed γ-glutamylation reactions 18

5. NMR spectra 19

5.1 *S*-allyl-L-cysteine (**2**) 19

5.2 γ-L-Glutamyl-*S*-allyl-L-cysteine (**3**) 19

6. HPLC chromatogram of the synthesis of γ-L-Glutamyl-*S*-allyl-L-cysteine (**3**) 20

Bibliography 21

1. Materials and Methods

1.1 General information

Calcium nitrate tetrahydrate, ammonium phosphate dibasic, allyl bromide, L-glutamine, L-glutamic acid, L-serine, L-cysteine hydrochloride monohydrate, L-glutamic acid 5-(*p*-nitroanilide) (GPNA), glycylglycine (GlyGly) and 1-fluoro-2,4-dinitrobenzene (Sanger's reagent) were from Merk KGaA (Darmstadt, Germany). All reagents were used as received without further purification. HPLC-grade solvents were from Aldrich.

GGT from *E. coli* was obtained as previously described.[1]

Experiments with hydroxyapatite of particle size between 180-250 μm were performed in a round bottom flask stirred using a rotary evaporator apparatus.

Continuous flow experiments were carried out using the ASIA pump system (Syrris Ltd, Royston, UK).

Analytical TLC was performed on silica gel F_{254} pre-coated aluminum sheets (0.2 mm layer) (Merck, Darmstadt, Germany). Eluent was a mixture of *n*-BuOH/water/AcOH 4:1:1. Detection: 5% w/v ninhydrin solution in ethanol, followed by heating at 150°C ca.

HPLC analyses were carried out using a 250 × 4.6 mm Gemini RP C18 column (Phenomenex, Torrance, CA, USA) on a Jasco instrument equipped with UV/Vis detector. Eluent A was 0.1% trifluoroacetic acid; eluent B was an 80:20 mixture of acetonitrile and eluent A. The following gradient was used: 0–10 min, isocratic elution with eluent A:eluent B 80:20; 10–15 min, linear gradient to eluent A:eluent B 70:30; 15–25 min, linear gradient to eluent A:eluent B 50:50; 25–35 min, linear gradient to eluent A:eluent B 40:60; 35– 40 min, isocratic elution with eluent A:eluent B 40:60; 40–60 min, column re-equilibration through linear gradient to eluent A:eluent B 80:20. Flow rate was 1 mL min^{-1} and detection was at 356 nm.

Ion exchange column chromatography was performed with Dowex 1×8 resin 200–400 20 mesh (Aldrich, Darmstadt, Germany) in the acetate form.

^{1}H-NMR spectra were acquired at 400.13 MHz on a Bruker Advance 400 spectrometer (Bruker, Karlsruhe, Germany) interfaced with a workstation running Windows operating system and equipped with a TOPSPIN software package. Chemical shifts are given in ppm (δ) and are referenced to solvent signal (δ_H D_2O 4.79 ppm). Spectra analyses were carried out with TOPSPIN software.

ATR-FTIR spectra were registered with a Jasco FT/IR-4600 spectrophotometer (Jasco International, Tokyo, Japan).

SEM images were obtained using a FESEM Sigma microscope (Zeiss).

XRPD analyses were carried out with a PANanalytical X'Pert PRO diffractometer (Cu Kα = 1.54060 Å) equipped with an X-ray source operating at 40 kV × 40 mA.

Measurements of surface area and pore size were performed by nitrogen adsorption-desorption using a Carlo Erba Sorptomatic 1990 instrument.

UV measurements were carried out with a Jasco V-360 Spectrophotometer (Jasco International, Tokyo, Japan).

No unexpected or unusually high safety hazard were encountered.

1.2 Hydroxyapatite synthesis and characterization

The synthesis of hydroxyapatite (HAP) was performed following a literature-reported method.[2]
Briefly, $(NH_4)_2HPO_4$ solution (0.1 M in degassed MilliQ water, 250 mL) was placed in a five-necked 1 L round bottom flask under nitrogen atmosphere and heated to 80°C and NH_4OH (30%, 50 mL) was added. Then, a $Ca(NO_3)_2 \bullet 4H_2O$ solution (0.167 M in degassed MilliQ water, 250 mL) was added under stirring through a peristaltic pump (flow rate 1.67 mL min^{-1}). A white solid started to precipitate.
Every 30 min, NH_4OH (30%, 10 mL) was added to maintain the pH basic. The reaction was stirred at 80°C for 2.5 h and then left ageing under stirring for 5 min.
Hot-filtration under reduced pressure was followed by washes with hot MilliQ water until the filtrate reached a neutral pH (ca. 4 x 500 mL). The obtained white solid was dried at 70°C under reduced

pressure (around 500 mBar) for 16 h and then at 120°C for 8 h. The dried solid was ground to obtain homogeneous particle size leading to a white powder.
To obtain larger particle sizes, the powder was first pressed using a hydraulic press; the resulting wafer was then ground and sieved using woven wire mesh stainless steel sieves by Endecotts, to obtain homogeneous particles in the size range of 180 - 250 μm.
The obtained hydroxyapatite was characterized via X-ray powder diffraction (XRPD), attenuated total reflection Fourier transform infrared (ATR-FTIR), scanning electron microscopy (SEM), and nitrogen adsorption-desorption analysis (BET method).

1.3 GGT expression and purification

GGT from *E. coli* was obtained from the wild-type *ggt* gene from strain BL21(DE3) genome (accession number CP001509) as previously described.[1] The gene was amplified using primers FGGTEcX (5′AAAACTCGAGGCCGCGCCTCCTGCGCCGCCC-3′) and RGGTEcB (5′-GGGGATCCTTAGTACCCCGCCGTTAAATCATC-3′), carrying the XhoI and BamHI restriction sites (underlined), respectively. The PCR product was restricted and cloned in the corresponding XhoI and BamHI sites of a pET16b expression plasmid to give plasmid pETGGT$_{Ec}$ which was verified by sequencing.
The recombinant protein was over-expressed and purified from *E. coli* BL21(DE3) cells as previously described.[3] Briefly, *N*-terminal His-tagged proteins were produced in autoinducing medium following the published protocol.[4] The cell pellet was resuspended in K buffer (50 mM Tris-HCl pH 7.5, 20 mM imidazole) and disrupted by sonication. After centrifugation, the cleared lysate was applied to Ni-agarose beads. After extensive washing with K buffer, proteins were eluted with the same buffer supplemented with 500mM imidazole. Purified GGTs were dialysed against 50mM Tris-HCl pH 7.5, 50mM NaCl, 30% Glycerol. Protein purity and efficiency of the autocatalytic cleavage were verified by SDS-PAGE analysis.[5] Aliquots were stored at −20 °C till use.

1.4 GGT activity assay

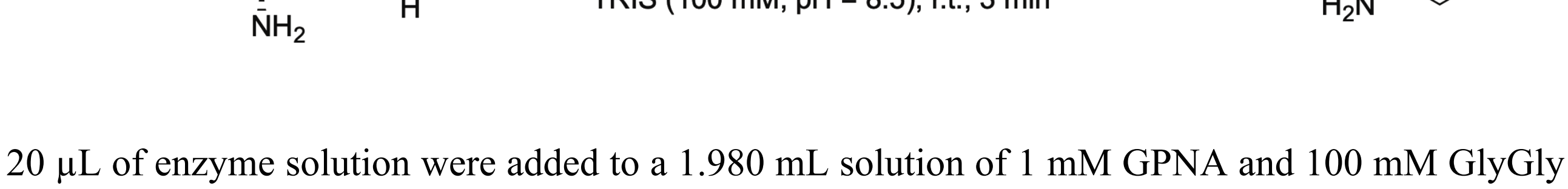

20 μL of enzyme solution were added to a 1.980 mL solution of 1 mM GPNA and 100 mM GlyGly in 100 mM TRIS buffer at pH 8.5 at room temperature. The release of *p*-nitroaniline was continuously

monitored at 410 nm, registering data every 10 s for 180 s. One enzyme unit was defined as the amount of enzyme that liberates 1 µmol mL^{-1} min^{-1} of *p*-nitroaniline. The concentration of *p*-nitroaniline was determined through a calibration curve (Figure S1).

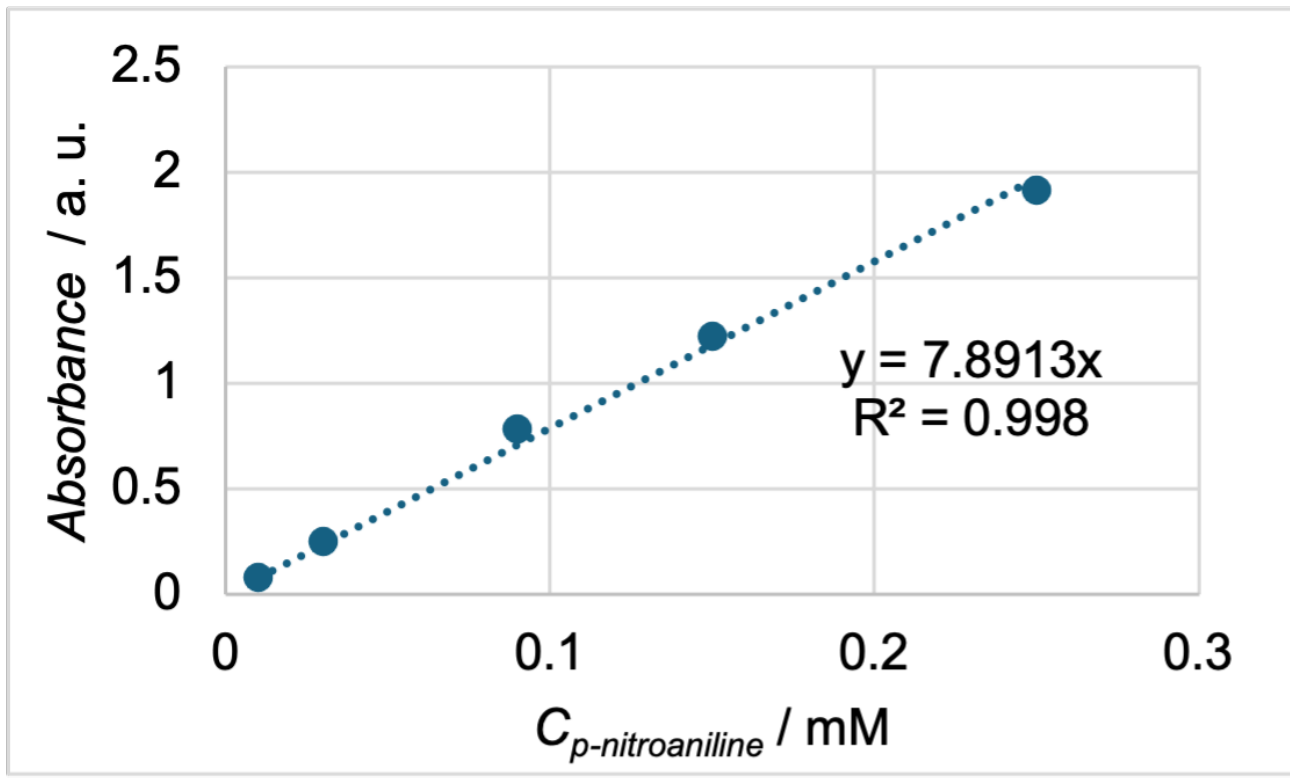

Figure S1. Calibration curve of *p*-nitroaniline

1.5 GGT immobilization

1.5.1 Conditions screening

Determination of favorable immobilization conditions was performed as follows: hydroxyapatite (50 mg) of different particle sizes, namely <200 nm or 180-250 µm, was suspended in an appropriate medium (100 mM carbonate/bicarbonate buffer (CBB); 100 mM TRIS, or deionized water) in the amount needed to have a final volume of 1.0 mL, at a certain pH (between 7 and 9.2) and thermostated at the desired temperature (0°C or 30°C). GGT from *Escherichia coli* (0.30 U, final supernatant activity 0.30 U mL^{-1}) was added and the suspension was stirred for 4 h.

The immobilization process was monitored by taking an aliquot (40 µL) of supernatant every hour and performing the enzymatic activity assay on it (see Paragraph 1.4).

The mixture was then separated by centrifugation (8000 rpm, 2 min, Universal 32 Hettich Zentrifugen) in the case of nanometric particles or by filtration under reduced pressure in the case of micrometric ones. The solid was washed with the immobilization medium (2 x 1 mL).

The enzyme concentration in both the filtrate and the washing was measured through Bradford assay and the residual activity was determined by activity assay. The final immobilization yield (IY) was determined as reported in Paragraph 1.5.4.

1.5.2 Loading screening

Hydroxyapatite (180-250 µm particle size) in a proper amount to achieve a maximum enzymatic loading of 2.5, 5.0 or 10 U g^{-1} was placed in a round bottom flask and suspended in TRIS buffer (100 mM, pH 8.5) in a quantity which, when added to the enzyme, will lead to 0.30 U mL^{-1}. The GGT solution (0.30 U, final supernatant activity 0.30 U mL^{-1}) was added and the suspension was stirred at 30°C for 7 h.

The immobilization process was monitored by performing the enzymatic activity assay on the supernatant (40 µL aliquot).

After 7 h the supernatant was removed with a Pasteur pipette and the solid was washed with TRIS buffer (100 mM, pH 8.5, 2 x 1 mL). The solid was tested to detect the immobilized enzymatic activity (see Paragraph 1.5.3).

1.5.3 Determination of the activity of the immobilized GGT

The GGT immobilized on HAP (180-250 µm particle size) was placed in a round bottom flask and suspended in TRIS buffer (100 mM, pH 8.5, 1.0 mL). GlyGly (200 mM in TRIS pH 8.5, 2.0 mL) and GPNA (4 mM in TRIS pH 8.5, 1.0 mL) solutions were added and the reaction was stirred for 25 min at room temperature. Ten times periodically (namely after 1, 2, 3, 4, 7, 9, 12, 15, 20 and 25 min) the supernatant (40 µL) was taken and diluted 1:20 in TRIS pH 8.5 and the absorbance at 410 nm was measured. From the obtained data it was possible to determine the activity of the immobilized GGT.

1.5.4 Calculation of immobilization parameters

The immobilization yield (*IY*) was calculated through the following formula[6]:

$$IY = \left(1 - \frac{Act_{filtr} + Act_{wash}}{Act_0}\right) x\ 100 \qquad \text{(S1)}$$

where Act_{filtr} (U) is the enzyme activity in the filtrate, Act_{wash} (U) is the enzyme activity in the washing of the immobilized enzyme, and Act_0 (U) is the enzyme activity given at the start of the immobilization.

The immobilization efficiency (*Eff*) was determined as follows:

$$Eff = \frac{Act_{obs\ imm}}{Act_{theor\ imm}} x\ 100 \qquad \text{(S2)}$$

where $Act_{obs\ imm}$ (U) is the activity of the immobilized enzyme measured as described in Paragraph 1.5.3, while $Act_{theor\ imm}$ (U) is the activity theoretically immobilized calculated from the immobilization yield:

$$Act_{theor\ imm} = IY\ x\ Act_0 \quad \text{(S3)}$$

The activity recovery (*Act R*) was obtained through the formula:

$$Act\ R = \frac{Act_{obs\ imm}}{Act_0}\ x\ 100 \quad \text{(S4)}$$

1.6 Immobilized GGT stability tests

1.6.1 Desorption tests

The immobilized enzyme was suspended in different media (1 mL), namely 100 mM CBB, 100 mM TRIS buffer, NaCl solution (between 100 mM and 1 M), 200 mM or 500 mM sodium citrate solution, at different pH (between 7 and 10); the mixture was stirred for 5 h at 30°C. Then the mixture was filtered under reduced pressure and the solid was washed with TRIS buffer (100 mM, pH 8.5, 2 x 1 mL). The enzyme desorption was determined by measuring the protein amount in the supernatant solution via Bradford assay.

1.6.2 Thermal stability

The immobilized GGT (20 mg), after determination of its immobilized activity ($Act_{obs\ imm}$), was suspended in TRIS buffer (100 mM, pH 8.5, 1.0 mL). The suspension was incubated under stirring for 1 h at different temperatures, namely 30, 40 and 50°C. Then, the mixture was incubated in a water bath at room temperature for 30 s, GlyGly (200 mM in TRIS pH 8.5, 2.0 mL) and GPNA (4 mM in TRIS pH 8.5, 1.0 mL) solutions were added and the residual activity was measured as described in Paragraph 1.5.3. The measured activity was compared to the one measured at 30°C.

1.6.3 Storage stability

The storage stability of the free GGT was tested by performing the activity assay, as reported in Paragraph 1.4, on a GGT solution (starting activity 0.3 U/mL) stored at 4°C over a 90-day period.

The immobilized activity of the enzyme was tested as described in Paragraph 1.5.3 after different periods, and up to 90 days, during which it was stored at 4°C.

1.6.4 Enzyme recyclability (activity assay)

The immobilized GGT (50 mg) was suspended in TRIS buffer (100 mM, pH 8.5, 2.5 mL) in a round bottom flask. GlyGly solution (200 mM in TRIS buffer pH 8.5, 5.0 mL) and GPNA solution (4 mM in TRIS buffer pH 8.5, 2.5 mL) were added and the reaction was stirred at room temperature for 10 min. Every minute, an aliquot of supernatant (100 µL) was taken and diluted 1:20 in TRIS buffer (100 mM, pH 8.5, 1.9 mL) and the absorbance at 410 nm was measured.
After 10 min the reaction was filtered under reduced pressure and the immobilized GGT was washed with TRIS buffer (100 mM, pH 8.5, 2 x 1 mL). Then it was put again in a round-bottom flask and the procedure was repeated for a total of six consecutive reactions.

1.7 Synthesis of γ-L-glutamyl-*S*-allyl-L-cysteine (3)

1.7.1 Synthesis of *S*-allyl-L-cysteine (1)

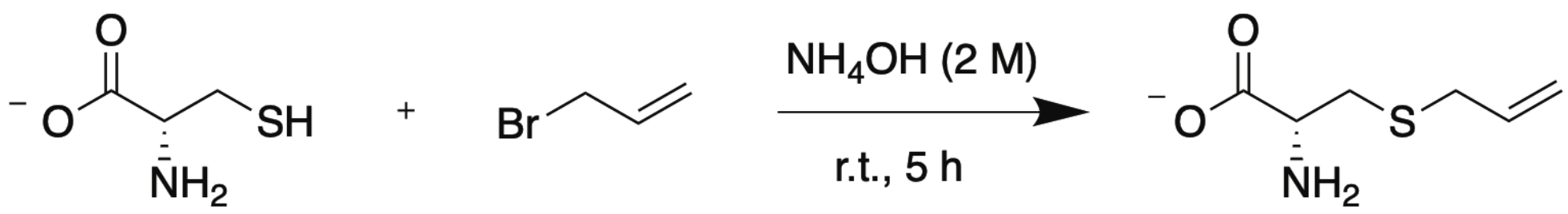

S-allyl-L-cysteine (**1**) was prepared as previously described.[7] Briefly, L-cysteine hydrochloride monohydrate (3.00 g, 17.1 mmol, 1 eq.) was dissolved in NH_4OH solution (2 M, 50 mL). Allyl bromide (1.78 mL, 20.5 mmol, 1.2 eq.) was added under stirring and the mixture was stirred at room temperature for 5 h. Then the mixture was concentrated under reduced pressure to around 1/5 of the initial volume; EtOH (15 mL) was then added and the resulting suspension was kept at 4°C overnight. The solid was collected by filtration under reduced pressure and washed with cold EtOH (2 x 10 mL) and Et_2O (10 mL).

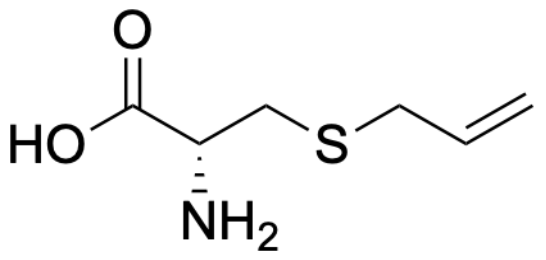

^{1}H-NMR (400 MHz, D_2O): δ 3.00 (dd, *J* = 14.8 Hz, 7.5 Hz, 1H), 3.11 (dd, *J* = 14.8 Hz, 4.3 Hz, 1H), 3.26 (d, *J* = 7.2, 2H), 3.95 (dd, *J* = 7.5 Hz, 3.4 Hz, 1H), 5.23 – 5.29 (m, 2H), 5.83 – 5.93 (m, 1H).

The NMR data are consistent with those reported in the literature.[7]

1.7.2 Enzymatic synthesis of γ-L-glutamyl-*S*-allyl-L-cysteine (3) and enzyme recyclability

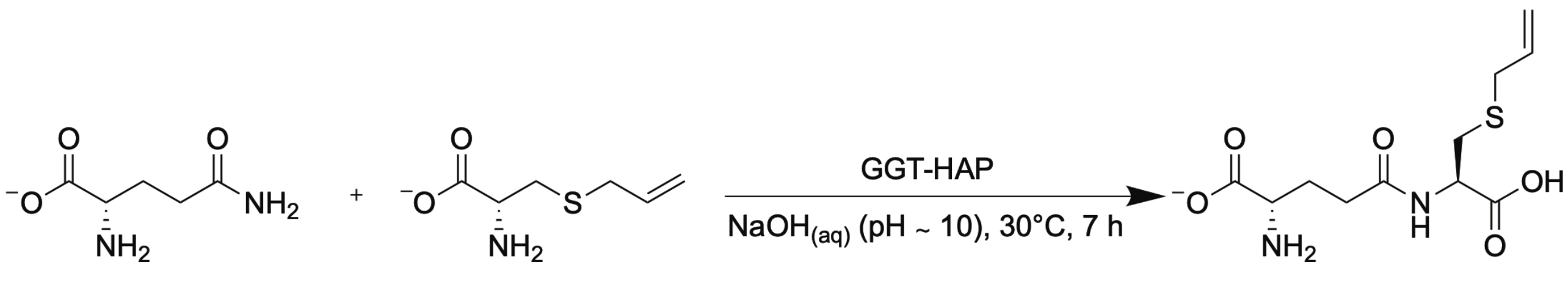

L-glutamine (Gln, 73.1 mg, 0.50 mmol) and *S*-allyl-L-cysteine (SAC, 80.6 mg, 0.50 mmol) were dissolved in deionized water and the pH was adjusted to 10 using an aqueous NaOH solution (1.0 M, final volume of the solution 5.0 mL). 2 mL of the resulting solution were added to the immobilized GGT (50 mg, 0.32 U). The reaction mixture was thermostated at 30°C and stirred for 7 h. Every hour the supernatant (20 µL) was taken and derivatized for HPLC analysis (see Paragraph 1.7.3).

After 7 h the supernatant was removed with a Pasteur pipette and the immobilized enzyme was washed with TRIS buffer (0.1 M, pH 8.5, 2 x 1 mL). The enzyme was stored at 4°C until the next reaction cycle. The enzyme was recycled 9 times.

1.7.3 Pre-column derivatization procedure with Sanger's reagent

Pre-column derivatization was carried out as already described.[8]

Briefly, standard solutions for calibration curve or aliquots of the reaction mixture (20 µL) were diluted 1:20 with water. 100 µL of the diluted solution was transferred into a Pyrex tube equipped with a perforated screw cap fitted with a forcible sealing septum. 50 µL of 5 mM L-serine in water was added as the internal standard, followed by 350 µL borate buffer (0.1 M, pH 8.5). The mixture was shaken and 500 µL of a 10 mM 1-fluoro-2,4-dinitrobenzene (Sanger's reagent) solution in acetone was added. The tube was sealed and heated at 70°C for 45 min in the dark. A needle was introduced into the septum and heating was continued for further 10 min, to evaporate acetone. The tube was cooled under running water and 200 µL of the resulting mixture was diluted 1:1 with 0.1% TFA solution before HPLC analysis.

1.7.4 Turnover number (TON) calculation

The turnover number (TON) of the immobilized GGT was calculated according to the following formula[9]:

$$TON = \frac{mmol\ of\ product\ formed}{mmol\ of\ enzyme} \qquad (S5)$$

Where the mmol of product formed are obtained from the reaction yield, while the mmol of immobilized enzyme are calculated by obtaining the mg of immobilized GGT through the Bradford assay and dividing them for the enzyme molecular weight (57.789 kDa).

1.7.5 Optimization of γ-L-glutamyl-*S*-allyl-L-cysteine (3) synthesis

The optimization of the reaction conditions was done by variation of the ratio of the reagents changing from 1:1 up to 1:3 Gln/SAC.

1.7.6 Semipreparative synthesis of L-γ-glutamyl-*S*-allyl-L-cysteine (3)

L-glutamine (Gln, 146.2 mg, 1.0 mmol) and *S*-allyl-L-cysteine (SAC, 483.7 mg, 3.0 mmol) were dissolved in deionized water (10 mL) and the pH was adjusted to 10 using an aqueous NaOH solution (1.0 M). The resulting solution was added to the immobilized GGT (250 mg, 1.0 U). The reaction mixture was thermostated at 30°C and stirred for 7 h.

Then the reaction was filtered under reduced pressure and the immobilized enzyme was washed with water (3 x 1 mL). The product contained in the filtrate was purified by ion exchange column chromatography: the mixture was charged onto a pad of Dowex 1×8 ion exchange resin in the acetate form and the pad was eluted with water (3 column volumes) and then with a scalar gradient of acetic acid solutions (0.5, 1.0, 1.5, and 2.0 M, three column volumes each). Eluate was collected in fractions; fractions were combined on the basis of TLC analysis (silica gel, eluent *n*-BuOH/AcOH/H_2O = 4:1:1, staining reagent: ninhydrin) and freeze-dried.

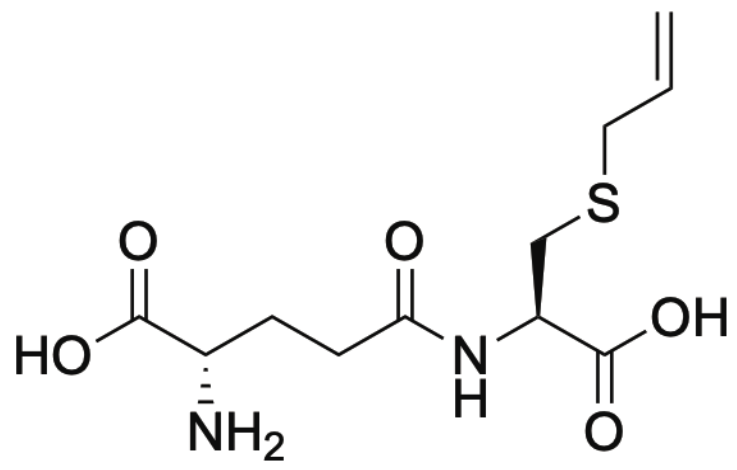

^{1}H-NMR (400 MHz, D_2O): δ 2.15 – 2.26 (m, 2H), 2.56 (t, *J* = 7.3 Hz, 2H), 2.90 (dd, *J* = 14.0 Hz, 7.9 Hz, 1H), 3.06 (dd, *J* = 14.0 Hz, 4.9 Hz, 1H), 3.24 (d, *J* = 7.1 Hz, 2H), 3.90 (t, *J* = 6.1 Hz, 1H), 4.56 (t, *J* = 6.4 Hz, 1H), 5.19 – 5.24 (m, 2H), 5.80 – 5.90 (m, 1H).

The NMR data are consistent with those reported in the literature.[7]

1.8 Continuous flow immobilization and synthesis

1.8.1 Determination of packed-bed reactor void volume

An Omnifit® column (height: 10 cm, empty volume 707 μL) was filled with sand (ca. 350 mg), hydroxyapatite (180-250 μm particle size, 200 mg) and sand again (ca. 300 mg). The reactor was

impregnated with TRIS buffer (100 mM, pH 8.5, flow rate 50 μL min^{-1}). Then a *p*-nitroaniline solution (1.5 mM in TRIS pH 8.5) was flowed through the reactor (flow rate 50 μL min^{-1}); the exiting solution was collected every minute in an Eppendorf vial. From every vial 40 μL of solution were withdrawn and diluted 1:20 with TRIS buffer (0.1 M pH 8.5) and the absorbance at 410 nm was measured producing a sigmoidal curve. The vial containing the solution with an absorbance higher than the first plateau was used to determine the reactor void volume as follows:

$$V_{without\ p-nitroaniline} = t_{exit}\ x\ flow\ rate \quad \text{(S6)}$$

$$V_{tot\ reactor} = V_{without\ p-nitroaniline} - V_{tubes} \quad \text{(S7)}$$

$$V_{void} = \frac{V_{tot\ reactor}\ x\ height_{HAP}}{height_{column}} \quad \text{(S8)}$$

Where $V_{without\ p\text{-}nitroaniline}$ is the volume of the solution which does not contain *p*-nitroaniline, calculated by multiplication of the time needed for the aniline solution to exit (t_{exit} in min which is equivalent to the number of collecting vial) and the flow rate in μL min^{-1}.

$V_{tot\ reactor}$ is the volume of liquid contained in the column and is calculated by subtracting the volume of the connecting tubes (V_{tubes}) from $V_{without\ p\text{-}nitroaniline}$.

Finally, V_{void} is the packed-bed reactor void volume, meaning the amount of solution present on the active part of the reactor (*i.e.* the hydroxyapatite on which the enzyme was immobilized). This parameter is calculated by multiplying the $V_{tot\ reactor}$ by the height of hydroxyapatite in the reactor ($height_{HAP}$) and dividing by the total height of the column ($height_{column}$).

The packed-bed reactor was washed with TRIS buffer (100 mM, pH 8.5).

1.8.2 Continuous flow adsorption

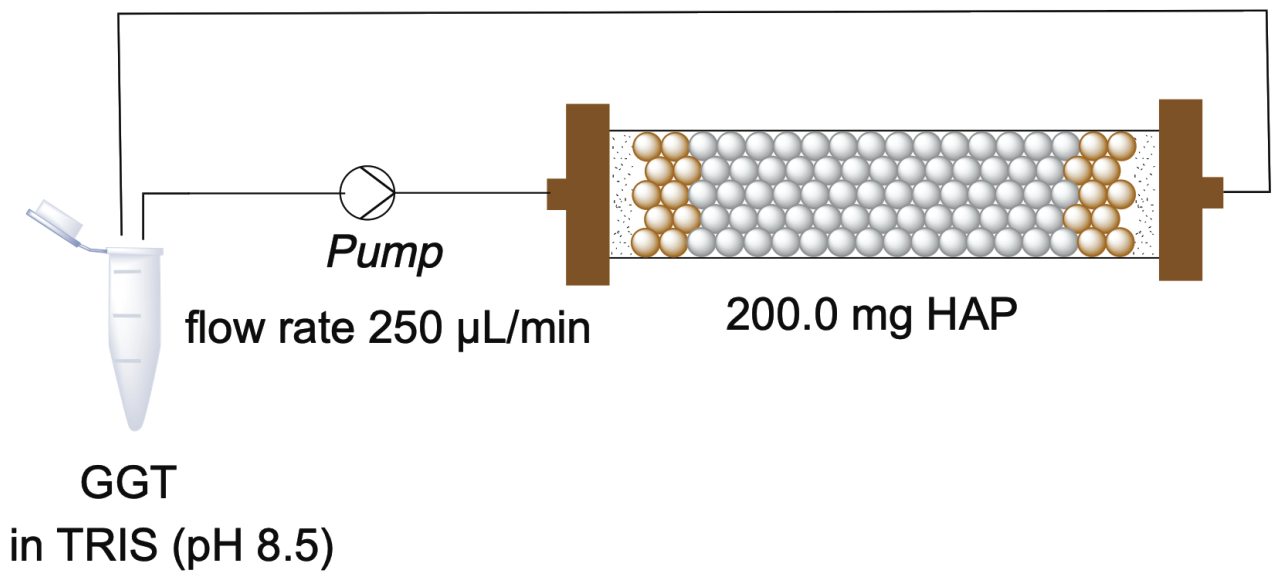

A solution of GGT in TRIS buffer (100 mM, pH 8.5) was recirculated through the packed-bed reactor (flow rate 250 µL min^{-1}) at room temperature. Different enzymatic activities (between 0.036 and 0.25 U mL^{-1}) and cycle numbers (6 or 8) were tested. The adsorption process was monitored by performing the enzymatic activity assay on the recirculating solution.
After the immobilization was completed, the packed-bed reactor was washed with TRIS buffer (100 mM, pH 8.5).

1.8.3 Residence time screening

A solution containing L-glutamine (**1**) and *S*-allyl-L-cysteine (**2**) (both 100 mM in $NaOH_{(aq)}$ pH 10) was flowed through the packed-bed reactor (2.5 U g^{-1}) with different residence times (between 1 and 20 min) at 30°C. For each residence time, six reactor volumes were collected and the last three were derivatized and analyzed by HPLC. Between each residence time experiment the packed-bed reactor was washed with TRIS buffer (100 mM, pH 8.5).

1.8.4 In flow synthesis of γ-L-glutamyl-*S*-allyl-L-cysteine (3)

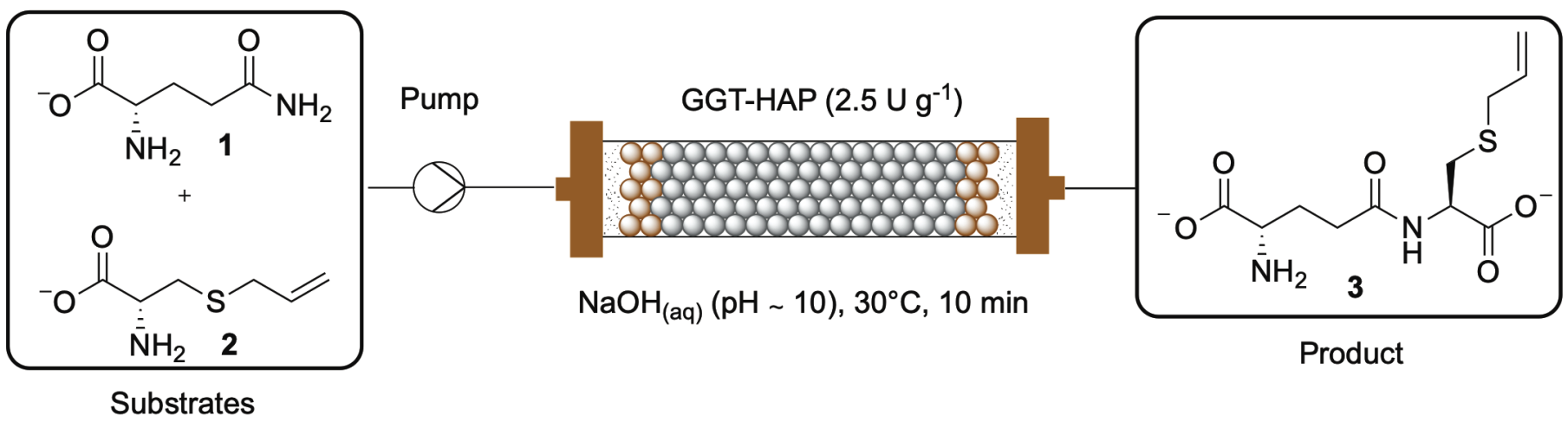

A solution containing L-glutamine (**1**, 100 mM) and *S*-allyl-L-cysteine (**2**) (either 100 mM or 300 mM) in $NaOH_{(aq)}$ (pH 10) was flowed through the packed-bed reactor (2.5 U g^{-1}) with a residence time of 10 min at 30°C. For each reaction, six reaction volumes were collected and the last three were derivatized and analyzed by HPLC.

1.8.5 Packed-bed recycle

A solution containing L-glutamine (**1**) and *S*-allyl-L-cysteine (**2**) (both 100 mM in $NaOH_{(aq)}$ pH 10) was flowed through the packed-bed reactor (2.5 U g^{-1}) with a residence time of 10 min at 30°C. For each reaction, six reaction volumes were collected and the last three were derivatized and analyzed by HPLC. The reactor was used to catalyze ten consecutive reactions. Between one reaction and the following, the reactor was cleaned by flowing TRIS (100 mM, pH 8.5, flow rate 250 µL min^{-1}) and stored at 4°C.

1.8.6 Calculation of productivity and space-time yield (STY)

Calculations to determine the productivity and the space-time yield (STY) of both the batch and the continuous flow set-ups are based on the data obtained from the first reactions performed using the following formulas, as previously reported in the literature[10]:

$$productivity = \frac{n_{prod}\ x\ \%yield}{t_{collection}\ x\ 100\%} \tag{S9}$$

Where $n_{product}$ (mmol) stands for the idealized quantitative yield of the product (*i.e.* the amount of product formed with a 100% yield), *%yield* is the HPLC yield and $t_{collection}$ (h) stands for the collection time until the idealized $n_{product}$ would be collected.

$$STY = \frac{n_R\ x\ \%yield}{V_R\ x\ t_{residence}\ x\ 100\%} \tag{S10}$$

In which n_R (mmol) are the mols present inside the reactor, *%yield* is the HPLC yield, V_R (mL) is the reactor volume (which in the case of the flow set-up corresponds to V_{void} as determined in Paragraph 1.8.1), and $t_{residence}$ (h) is the residence time.

2. Analyses of pristine HAP and GGT-HAP

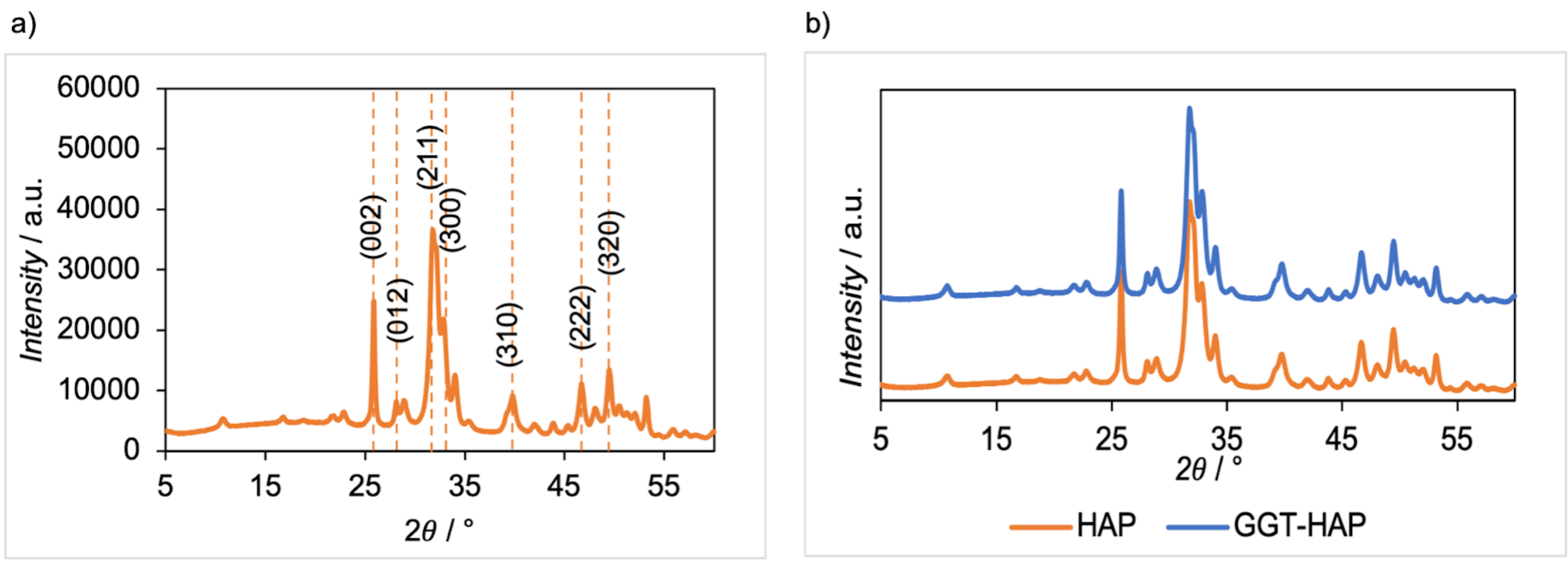

Figure S2. a) XRPD diffractogram of pure hydroxyapatite. b) Comparison between the XRPD diffractograms of pristine HAP (orange) and GGT-HAP (blue). No significant differences are present between the two, suggesting that the enzyme adsorption does not influence the crystalline lattice of the solid.

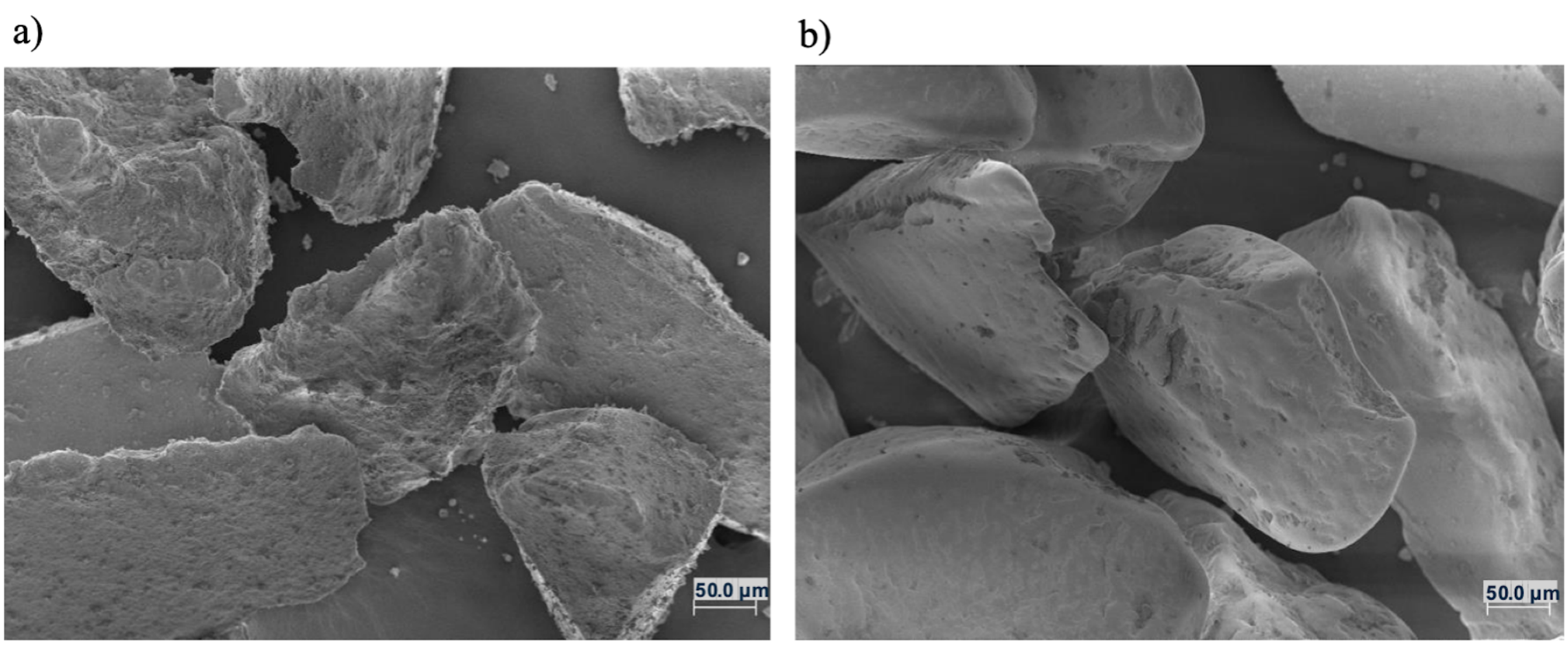

Figure S3. SEM image of: a) pure hydroxyapatite (HAP), and b) GGT-HAP

Table S1 FTIR band positions and assignment in HAP and GGT-HAP samples

Band (cm^{-1})[a]	Assignment	Reference
Bands shared by HAP and GGT-HAP		
558, *m*	ν_4 PO_4, P – O bending mode	[11,12]
600, *m*	ν_4 PO_4, P – O bending mode	[11,12]
881	ν_2 CO_3, out-of-plane bending modes of carbonates	[11–13]
963, *w*	ν_1 PO_4, symmetric P – O stretching mode	[11,12]
1020, *vs*	ν_3 PO_4, antisymmetric P – O stretching mode	[11,12]
1089, *s*	ν_3 PO_4, antisymmetric P – O stretching mode	[11,12]
1420	ν_2 CO_3 type B, antisymmetric stretching modes of carbonates substituting phosphates	[11–14]
1454	ν_2 CO_3 type A, antisymmetric stretching modes of carbonates substituting columnar OH^-	[11–14]
1493	both carbonates substituting columnar OH- (A-type) and carbonates substituting phosphates (B-type)	[11–14]
1648, *w*	δ H_2O, H_2O deformation mode	[11,12,14]
3569, *w*	ν_s OH stretching mode of bulk OH^-, occupying the 4e position in the hexagonal lattice ("columnar OH")	[11,12,14]
Bands unique to GGT-HAP		
720-1100	γ_r, rocking vibration, couples with adjacent CH_2 groups, in phase mode at 754 cm^{-1} most intense	[15,16]
1200-1400	amide III band (ν C – N, δ N – H)	[15,16]
1550	amide II band (N – H bending and C – N stretching of peptide backbone)	[15,16]
1650	amide I band (predominantly C=O stretching of peptide backbone)	[15,16]
2800-3000	C – H alkyl stretching	[15,16]
3070-3100	amide B band (ν N – H)	[15,16]
3100-3600	amide A band (ν N – H)	[15,16]

[a] *m: medium, s: strong, vs: very strong, w: weak*

3. Thermal stability of free *vs* immobilized GGT

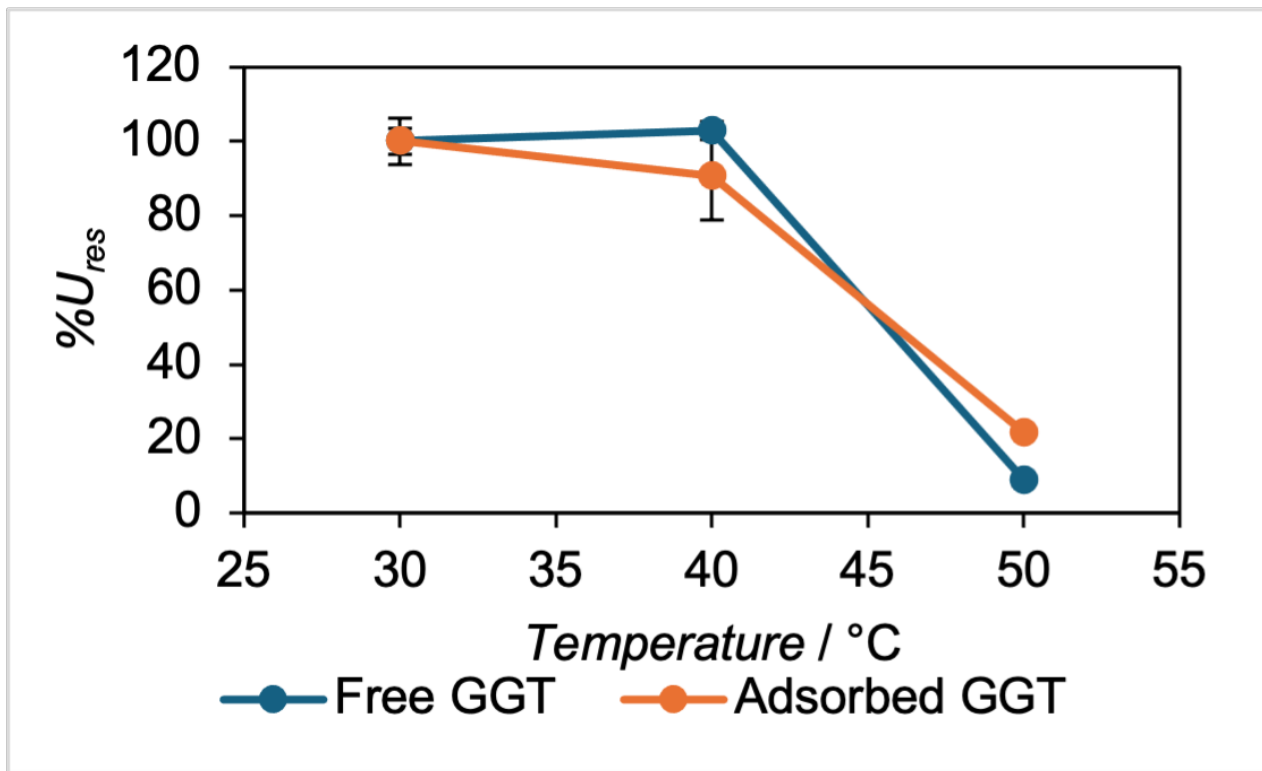

Figure S4. GGT thermal stability in free (blue) *vs* immobilized (orange) form. The residual activities at 40 and 50°C are referred to the activity at 30°C considered as 100%.

4. Reaction mechanism of GGT-catalyzed γ-glutamylation reactions

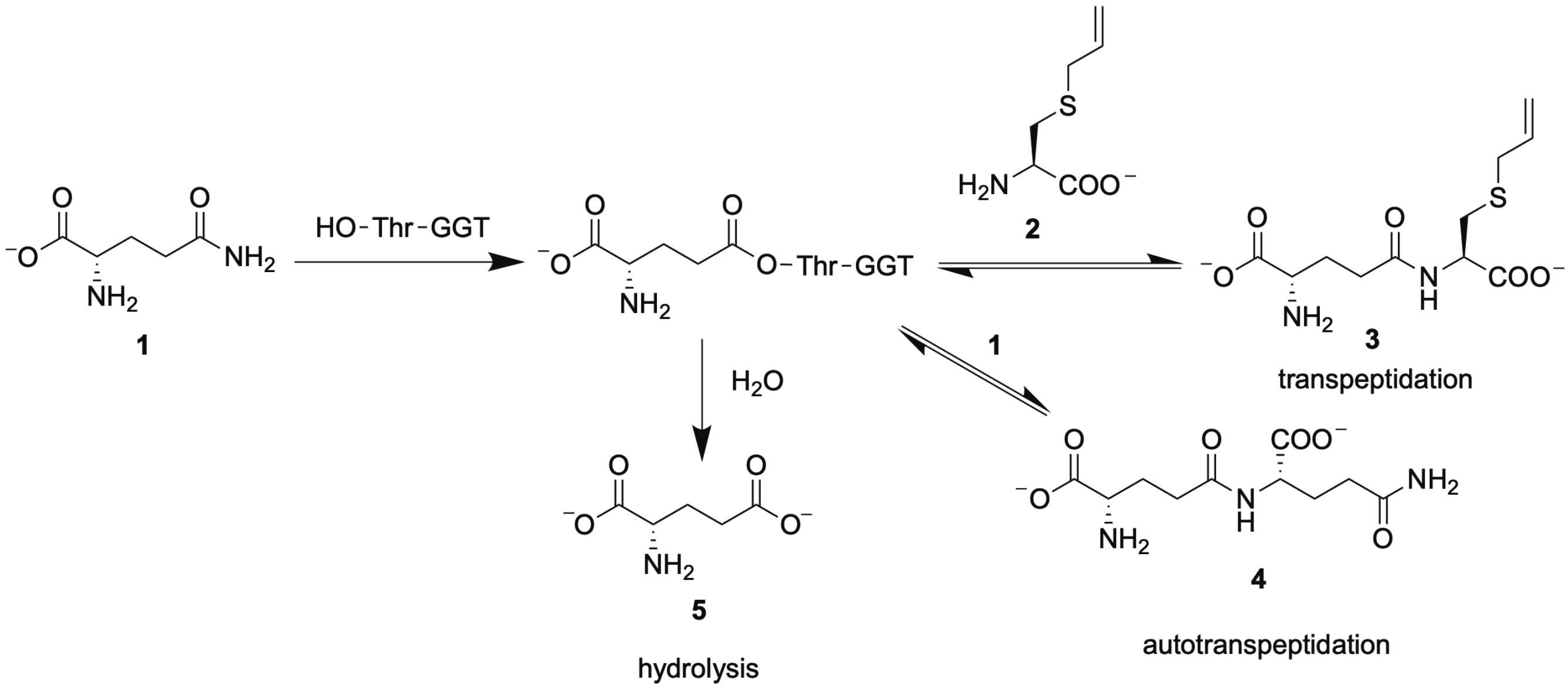

Scheme S1. Reaction mechanism of GGT catalyzed γ-glutamylation. It has to be noted that the only irreversible reaction occurring from the glutamylated enzyme intermediate is the hydrolysis, leading to **5**. This is the main reason why yields higher than 30-35% are not achievable with a donor/acceptor ratio of 1:1.

5. NMR spectra

5.1 *S*-allyl-L-cysteine (2)

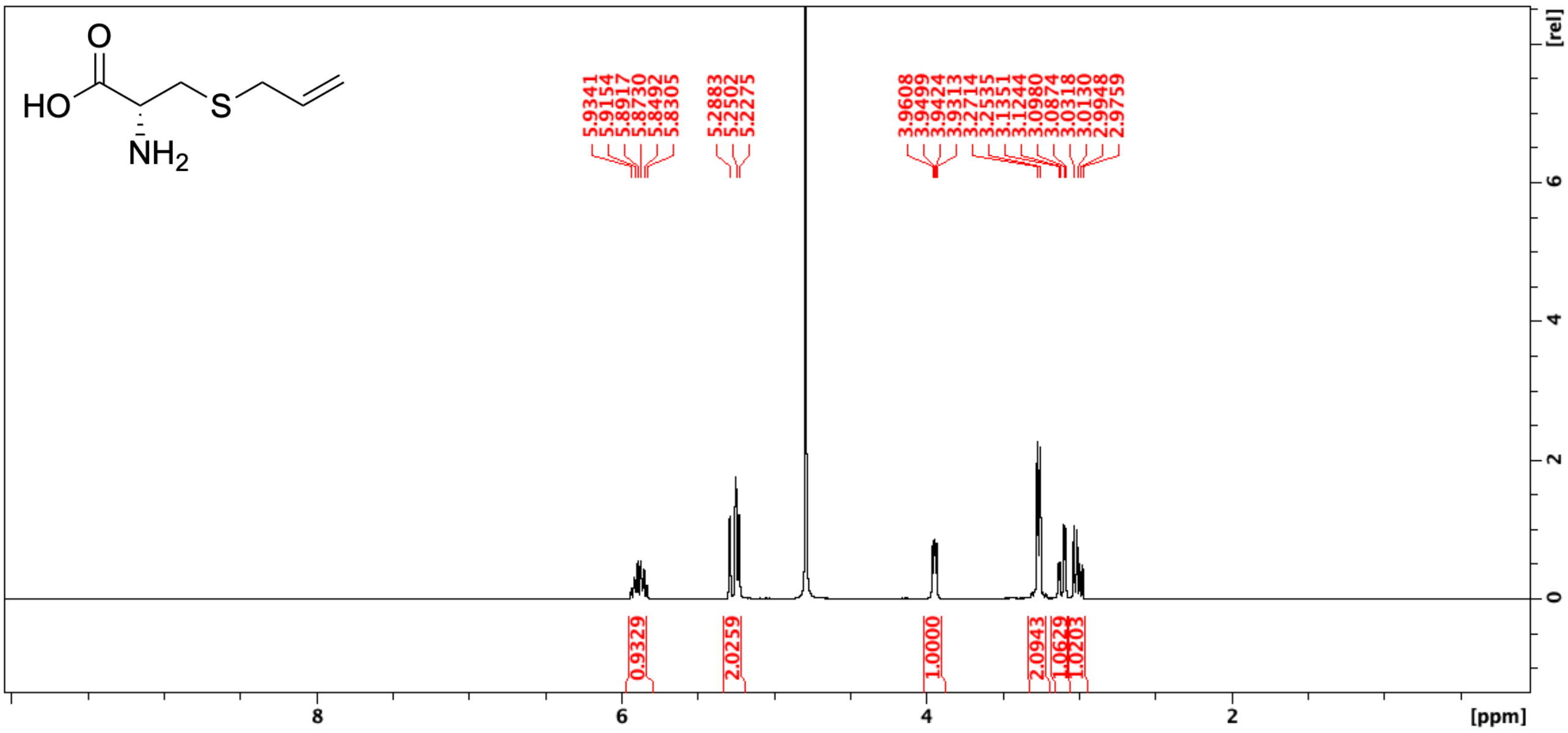

5.2 γ-L-Glutamyl-*S*-allyl-L-cysteine (3)

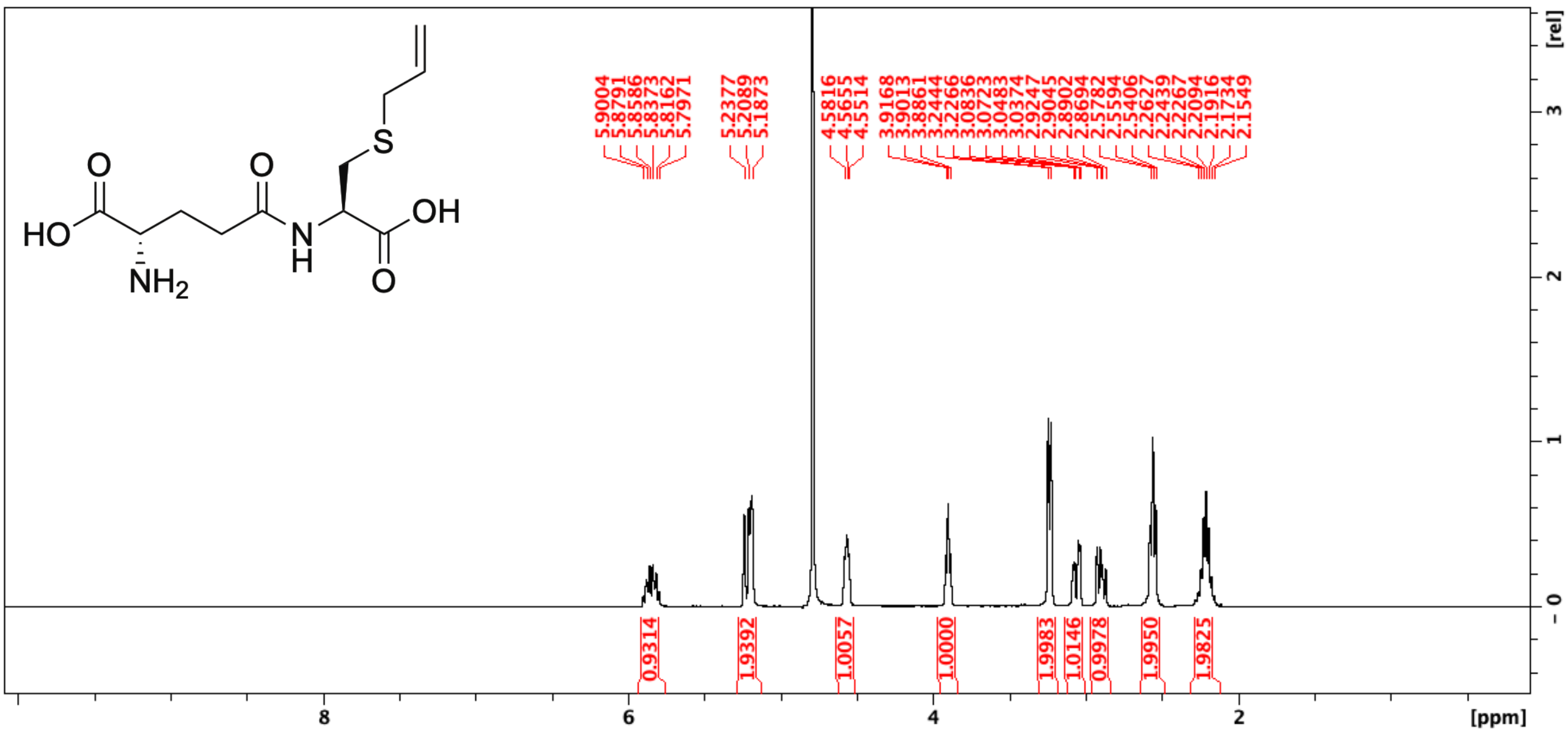

6. HPLC chromatogram of the synthesis of γ-L-Glutamyl-*S*-allyl-L-cysteine (3)

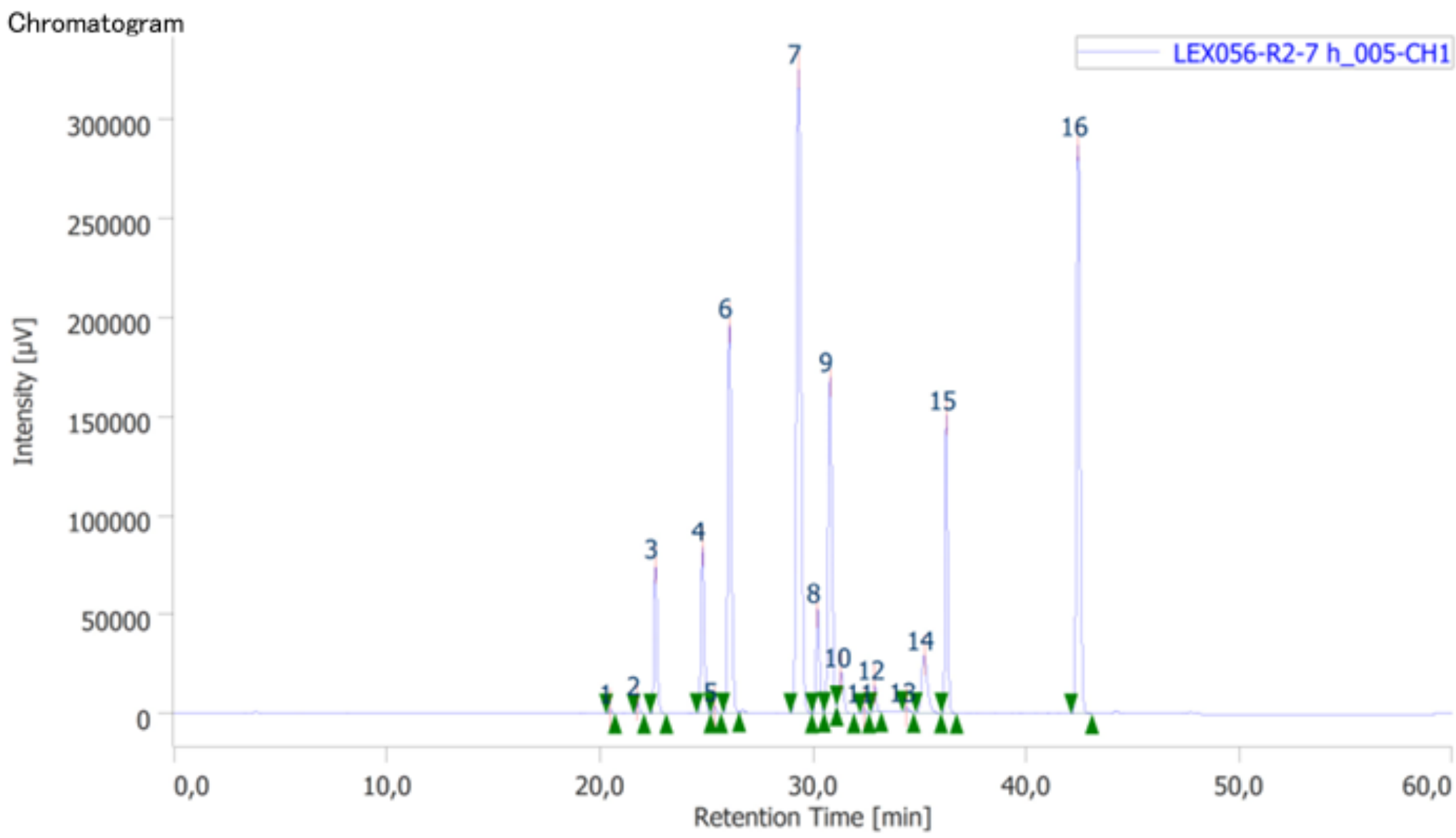

Peaks attribution: 2) γGlu-γGlu-Gln; 3) γGluGln; 4) Gln; 6) Ser, used as internal standard; 7) excess Sanger's reagent; 8) Glu; 9) 2,4-dinitroaniline, arising from the reaction of Sanger's reagent with ammonia liberated from Gln; 10) derivatization byproduct; 12) γGlu-γGlu-SAC; 14) derivatization byproduct; 15) γ-Glu-SAC; 16) SAC.

The reported chromatogram was obtained for the reaction with a reagent ratio of 1:1.

Bibliography

(1) Calvio, C.; Romagnuolo, F.; Vulcano, F.; Speranza, G.; Morelli, C. F. Evidences on the Role of the Lid Loop of γ-Glutamyltransferases (GGT) in Substrate Selection. *Enzyme Microb. Technol.* **2018**, *114*, 55–62. https://doi.org/10.1016/j.enzmictec.2018.04.001.

(2) Campisi, S.; Castellano, C.; Gervasini, A. Tailoring the Structural and Morphological Properties of Hydroxyapatite Materials to Enhance the Capture Efficiency towards Copper(II) and Lead(II) Ions. *New Journal of Chemistry* **2018**, *42* (6), 4520–4530. https://doi.org/10.1039/c8nj00468d.

(3) Morelli, C. F.; Calvio, C.; Biagiotti, M.; Speranza, G. PH-Dependent Hydrolase, Glutaminase, Transpeptidase and Autotranspeptidase Activities of Bacillus Subtilis γ-Glutamyltransferase. *FEBS Journal* **2014**, *281* (1), 232–245. https://doi.org/10.1111/febs.12591.

(4) Studier, F. W. Protein Production by Auto-Induction in High Density Shaking Cultures. *Protein Expr. Purif.* **2005**, *41* (1), 207–234. https://doi.org/10.1016/j.pep.2005.01.016.

(5) Chi, M. C.; Lo, Y. H.; Chen, Y. Y.; Lin, L. L.; Merlino, A. γ-Glutamyl Transpeptidase Architecture: Effect of Extra Sequence Deletion on Autoprocessing, Structure and Stability of the Protein from Bacillus Licheniformis. *Biochim. Biophys. Acta Proteins Proteom.* **2014**, *1844* (12), 2290–2297. https://doi.org/10.1016/j.bbapap.2014.09.001.

(6) Sheldon, R. A.; van Pelt, S. Enzyme Immobilisation in Biocatalysis: Why, What and How. *Chem. Soc. Rev.* **2013**, *42* (15), 6223–6235. https://doi.org/10.1039/c3cs60075k.

(7) Speranza, G.; Rabuffetti, M.; Vidović, N.; Morelli, C. F. Synthesis of γ-Glutamyl Derivatives of Sulfur-Containing Amino Acids in a Multigram Scale via a Two-Step, One-Pot Procedure. *Molbank* **2020**, *2020* (3), 1–8. https://doi.org/10.3390/M1147.

(8) Rabuffetti, M.; Speranza, G.; Calvio, C.; Morelli, C. F. Enzymatic Synthesis of γ-Glutamyl Dipeptides Catalysed by Mutant E. Coli γ-Glutamyltransferases. *European J. Org. Chem.* **2022**, *2022* (43), e202200907. https://doi.org/10.1002/ejoc.202200907.

(9) Faber, K. *Biotransformations in Organic Chemistry*, 7th ed.; Springer Cham: Cham, 2018.

(10) Herbrik, F.; Sanz, M.; Puglisi, A.; Rossi, S.; Benaglia, M. Enantioselective Organophotocatalytic Telescoped Synthesis of a Chiral Privileged Active Pharmaceutical Ingredient. *Chemistry - A European Journal* **2022**, *28* (33), e202200164. https://doi.org/10.1002/chem.202200164.

(11) Brown, P. W.; Constantz, B. *Hydroxyapatite and Related Materials*; CRC Press, 2017. https://doi.org/10.1201/9780203751367.

(12) Rey, C.; Marsan, O.; Combes, C.; Drouet, C.; Grossin, D.; Sarda, S. Characterization of Calcium Phosphates Using Vibrational Spectroscopies. In *Advances in Calcium Phosphate Biomaterials*; Ben-Nissan, B., Ed.; Springer: Berlin, Heidelberg, 2014; Vol. 2, pp 229–266. https://doi.org/10.1007/978-3-642-53980-0_8.

(13) Fleet, M. E. *Carbonated Hydroxyapatite: Materials, Synthesis, and Applications*, 1st edition.; Jenny Stanford Publishing: New York, 2014. https://doi.org/10.1201/b17954.

(14) Sakhno, Y.; Ivanchenko, P.; Iafisco, M.; Tampieri, A.; Martra, G. A Step toward Control of the Surface Structure of Biomimetic Hydroxyapatite Nanoparticles: Effect of Carboxylates on the {010} P-Rich/Ca-Rich Facets Ratio. *The Journal of Physical Chemistry C* **2015**, *119* (11), 5928–5937. https://doi.org/10.1021/jp510492m.

(15) Barth, A. Infrared Spectroscopy of Proteins. *Biochimica et Biophysica Acta (BBA) - Bioenergetics* **2007**, *1767* (9), 1073–1101. https://doi.org/10.1016/j.bbabio.2007.06.004.

(16) Krimm, S. Interpreting Infrared Spectra of Peptides and Proteins. In *Infrared Analysis of Peptides and Proteins*; Singh, B. R., Ed.; American Chemical Society, 1999; Vol. 750, pp 38–53. https://doi.org/10.1021/bk-2000-0750.ch002.
